# Lowering of innate resistance of the lungs to the growth of blood-borne cancer cells in states of topical and systemic stress.

**DOI:** 10.1038/bjc.1976.7

**Published:** 1976-01

**Authors:** H. A. Van Den Brenk, M. G. Stone, H. Kelly, C. Sharpington

## Abstract

The survival and clonogenic growth (measured in terms of colony forming efficiency (CFE) of intravenously injected (i.v.) Walker (W256) tumour cells in the lungs of rats was greatly enhanced by states of topical and systemic stress induced by the intraperitoneal (i.p.) injection of rats with a single dose of 10(-5)-10(-3) mmol g-1 body weight of adrenaline and other beta-adrenergic agonists, inflammatory agents (including local x-irradiation), convulsive seizures, "tumbling" or physical restraint. Lowering of innate resistance of the host to growth of seeded tumour cells induced by states of topical and systemic stress, and by the addition of an excess of lethally irradiated (LI) tumour cells to i.v. injected intact tumour cells, were all potentiated by treatment of rats with aminophylline, an inhibitor of cyclic AMP phosphodiesterase. Enhancement of tumour growth by systemic stress was inhibited by bilateral total or medullary adrenalectomy and is attributed to the release and actions of endogenous adreno-medullary hormones. Alpha-adrenergic and most non-adrenergic agents administered in maximum tolerated doses did not significantly affect host resistance to tumour growth in the lungs. These findings, correlated with measurements of cyclic AMP in the lungs of normal and stressed rats, suggest that changes in the resistance of the host to tumour growth involve changes in cyclic nucleotide metabolism in the target tissues (tumour bed); possible mechanisms of action of cyclic nucleotides in this respect are discussed.


*Br. J. Cancer* (1976) **33**, 60

### LOWERING OF INNATE RESISTANCE OF THE LUNGS TO THE GROWTH OF BLOOD-BORNE CANCER CELLS IN STATES OF TOPICAL AND SYSTEMIC STRESS

H. A. S. VAN DEN BRENK, M. G. STONE, H. KELLY AND C. SHARPINGTON

*From the Richard Dimbleby Cancer Research Laboratory, St Thomas' Hospital, London SE1 7EH*

Received 14 August 1975 Accepted 26 September 1975

**Summary.—The survival and clonogenic growth (measured in terms of colony forming efficiency (CFE)) of intravenously injected (i.v.) Walker (W256) tumour cells in the lungs of rats was greatly enhanced by states of topical and systemic stress induced by the intraperitoneal (i.p.) injection of rats with a single dose of $10^{-5}$–$10^{-3}$ mmol $g^{-1}$ body weight of adrenaline and other $\beta$-adrenergic agonists, inflammatory agents (including local x-irradiation), convulsive seizures, "tumbling" or physical restraint. Lowering of innate resistance of the host to growth of seeded tumour cells induced by states of topical and systemic stress, and by the addition of an excess of lethally irradiated (LI) tumour cells to i.v. injected intact tumour cells, were all potentiated by treatment of rats with aminophylline, an inhibitor of cyclic AMP phosphodiesterase. Enhancement of tumour growth by systemic stress was inhibited by bilateral total or medullary adrenalectomy and is attributed to the release and actions of endogenous adreno-medullary hormones. Alpha-adrenergic and most non-adrenergic agents administered in maximum tolerated doses did not significantly affect host resistance to tumour growth in the lungs.**

**These findings, correlated with measurements of cyclic AMP in the lungs of normal and stressed rats, suggest that changes in the resistance of the host to tumour growth involve changes in cyclic nucleotide metabolism in the target tissues (tumour bed); possible mechanisms of action of cyclic nucleotides in this respect are discussed.**

ENHANCED growth of intravascularly injected tumour cells has been shown to occur in injured tissues of organs of rats (Fisher and Fisher, 1959*a*, *b*, 1962; Robinson and Hoppe, 1962; Alexander and Altmeier, 1964). Clonogenic growth of i.v. injected tumour cells in the lungs and other organs of rats and mice is markedly enhanced by prior x-irradiation of the target organ (Withers and Milas, 1973; Brown, 1973; van den Brenk *et al.*, 1973*a*; van den Brenk and Kelly, 1973, 1974). Chemically induced inflammatory reactions similarly enhance tumour colony forming efficiency (CFE) in the lungs of rats (van den Brenk *et al.*, 1974). In these various investigations, suggestive but not clear-cut evidence was obtained that tumour growth was stimulated by systemic stress induced in the animal by tissue injury; it was essentially attributed to local pathophysiological changes produced in the target organ by the injurious agent. However, the studies of Fisher and Fisher (1959*b*) did indicate that systemic stress affected tumour growth since a laparotomy stimulated growth of tumour in the liver of rats. Recently, Peters (1975) has reported that laparotomy enhanced take and growth of subcutaneously injected tumour cells in mice. These findings suggest that systemic stress indirectly lowers an innate resistance of tissues to growth of cancer cells. Presumably this results from pharmacologically induced changes in the target tissue which are mediated *via* neuroendocrinal pathways. This common property of agents, which cause topical or systemic stress, of lowering resistance of tissues to tumour growth under appropriate conditions has caused us to investigate the effects of $\beta$-adrenergic agents on CFE and the possibility that a common biochemical mechanism of action is involved which is fundamentally associated with the perturbation of cyclic nucleotide metabolism in the tumour bed. The release of adrenaline, its activation of $\beta$-adrenergic receptor bound adenyl cyclase (AC) and stimulation of cyclic adenosine-3′-5′-monophosphate (c-AMP) in a variety of tissues, including lung (Robinson, Butcher and Sutherland, 1971; Jost and Rickenberg, 1971) plays a principal role in the physiological changes seen in states of stress. We have compared the effects on tumour CFE in the lungs of treating rats with $\beta$-adrenergic agonists with that of a variety of other pharmacologically active drugs, and similarly studied the effects on tumour growth of aminophylline, an inhibitor of phosphodiesterase (PD), the enzyme which causes metabolic degradation of c-AMP in tissues. The effects of chemically and physically induced systemic stress on tumour CFE and measurements of c-AMP levels in irradiated and unirradiated lungs of rats are described.

### MATERIALS AND METHODS

The lung colony assay technique, using a subline of Walker (W256) tumour cells, prepared in single cell suspension and injected i.v. in SPF Caworth Farm strain rats, has been described previously (van den Brenk, Sharpington and Orton, 1973*b*). Tumour CFE was determined 8 days after the injection of tumour cells as, CFE $= N_L/N$ where $N_L$ is the number of lung tumour macrocolonies produced on the surfaces of the lungs of a rat by the i.v. injection of $N$ tumour cells. The effect on CFE of treating rats with a particular agent was measured as the ratio $R(N_L) = CFE(T)/CFE(C)$ for 2 groups of 6–8 treated (T) rats and 6–8 control (C) rats of the same age which had been injected i.v. with the same number of tumour cells from the same tumour cell suspension. The significance of R was calculated by Student's "t" test with Bessel's correction. Since increases in the number of 8-day old tumour colonies per lung were shown to cause proportional increases in lung wet weight, a corresponding ratio $R(w)$ based on lung weight was calculated provided the treatment with the agent alone caused no significant change in lung weight of rats (measured per unit body weight) 8 days later. It was difficult to count accurately lung colonies in excess of 200 per rat; in such experiments $R(w)$ provided the better basis for comparisons of tumour growth. Since tumour CFE depends very greatly on age of rat, rats of approximately the same age (within a range of 5 days) were selected for each treated and corresponding control group. Each rat was weighed before it was injected with tumour cells and again when killed to measure tumour growth 8 days later.

### *Drugs and dosages*

The following drugs were used: o-Acetylcholine chloride (Hopkin & Williams Ltd), adenosine-3′-5′-monophosphate cyclic (Hopkin and Williams Ltd), angiotensin amide (Hypertensin, CIBA Laboratories Ltd.), atropine sulphate, bradykinin (BRS 640, Sandoz Ltd.), bretylium tosylate (Darenthin, Burroughs Wellcome and Co.), 2-bromolysergic acid diethylamide tartrate (BOL 148, Sandoz Ltd), cyclophosphamide monohydrate (Endoxana, W. B. Pharmaceuticals Ltd), $N^6,O^{2'}$-dibutyryl adenosine-3′-5′-cyclic phosphoric acid sodium salt (d,b,-cAMP, BDH Chemicals Ltd), glucagon (Eli Lilly & Co. Ltd), guanethidine sulphate (Ismelin, CIBA Laboratories Ltd), histamine acid phosphate (BDH Chemicals Ltd), 5-hydroxytryptamine creatinine sulphate (serotonin creatinine sulphate, BDH Chemicals Ltd), imidazole (BDH Chemicals Ltd), isopropyl noradrenaline sulphate (isoprenaline, Burroughs Wellcome & Co.), mepyramine maleate (Anthisan, May & Baker Ltd), N- (2- (3, 4- methylenedioxyphenylisopropyl)) noradrenaline (Protokylol, Lakeside Laboratories), N-methyl-noradrenaline (adrenaline, Hopkin & Williams Ltd), L-noradrenaline hydrochloride (L-arterenol, Sigma Chemical Co.), pentobarbitone sodium (Nembutal, Abbott Laboratories Ltd), pentylenetetrazole (Leptazol, Martindale Samoore Ltd), phenoxybenzamine hydrochoride (Dibenyline, Smith Kline & French Laboratories

Ltd), practolol (ICI Ltd), propranolol (ICI Ltd), prostaglandins A, $E_1$, $E_2$, $F_{2\alpha}$ (Upjohn Ltd), sodium acetazolamide (Diamox, Lederle Laboratories Ltd), sodium ethacrynate (Edecrin, Merck Sharp and Dohme Ltd), theophylline ethylene diamine (aminophylline, Antigen Ltd), theophylline hydrate (BDH Chemicals Ltd, vasopressin (Pitressin, Parke-Davis & Co.). All other chemicals were analytical grade. $^3$H-thymidine was supplied by the Radiochemical Centre, Amersham, and Compound 48/80 was kindly donated by Wellcome Research Laboratories. Cellulose sulphate was prepared as described previously (van den Brenk *et al.*, 1974).

Unless stated otherwise, the drugs were injected intraperitoneally (i.p.) in single doses, expressed in terms of mmol drug/g body weight. Each agent was tested for acute toxicity; the "maximum tolerated" dosage used in tumour injected rats did not exceed one-half of the $LD_{50}$ dose or $10^{-4}$ mmol drug/g body weight. Signs of prostration and stress occurred within a few min after injection of $\beta$-adrenergic and other agents in "maximum tolerated" dosages but the rats recovered relatively rapidly. Tumour CFE was measured only in those experiments in which at least 80% of rats had survived and gained weight at a mean rate of not less than 2 g/day for 8 days after the injection of the drug and the tumour cells.

*Local x-irradiation of the thorax* (*LTI*)

A single dose of 1000 rad to the whole thorax or hemithorax with the remainder of the body of the rat shielded was given under pentobarbitone sodium anaesthesia 7 days before injection of the tumour cells, as described previously (van den Brenk *et al.*, 1973*a*).

*Adrenalectomy*

Bilateral total ($TA_X$) or medullary ($MA_X$) adrenalectomy was performed and replacement therapy given as described previously (van den Brenk *et al.*, 1974).

*Systemic stress*

*Techniques.*—Three methods of stressing rats were used:

(1) *Chemical stress—convulsive seizures.*—Six-week old rats were injected i.p. with a single dose of 1·2–12·5 mg pentylenetetrazole (PTZ), an agent which induces seizures in rats. The seizures are violent and sustained (grand mal) and prove fatal within 5–10 min in unanaesthetized rats when the dose of PTZ exceeds 5 mg; a lower dose of 3 mg PTZ causes slight twitchings (petit mal), a dose of 5 mg PTZ causes grand mal seizures from which approximately 80% of rats recover and survive. In rats which have been anaesthetized with 38 mg pentobarbitone sodium/kg body weight the injection of 5 mg PTZ causes an earlier awakening of the rats than usual but no seizures develop and all rats survive. An increase in the dose to 12·5 mg PTZ in anaesthetized rats causes rapid awakening and approximately 50% rats develop grand mal seizures and the remainder petit mal attacks, but there are no fatalities. Tumour CFE was measured under these various conditions of treatment with PTZ given 5–10 min *after* the i. v. injection of tumour cells.

(2) *Physical stress.*—(a) *Tumbler technique.*—To cause stress, a group of rats was placed in a closed cylindrical drum which could be rapidly rotated about its central axis on a horizontal rod held clamped in a vice. The drum was fitted with a fixed internal vane which tumbled the rats inside the drum as it was spun by hand at a rate of about 2 rev/s changing from clockwise to anti-clockwise every 30 s. The stress of tumbling was supplemented by that of loud noise produced by an assistant hammering a metal rod; the noise intensity was not measured. The rats were tumbled for three 5-min periods separated by 2 rest periods of 1 h each. Tumour CFE was measured in rats which were tumbled 24 h before, 30 min after, or 24 h after i. v. injection of tumour cells. (b) *Stress immobilization.*—The technique of Selye (1954) was used to stress rats by taping conscious rats to a wooden lathe with Sellotape for a total time of 3 h. The rats were taped head down on to the board which was held at an angle of about 80° to the horizontal plane. This procedure induced struggling but prevented escape. Immobilization was commenced 30 min *after* i.v. injection of intact or adrenalectomized rats with tumour cells.

*Assay of adenosine-3′-5′-monophosphate cyclic* (*c-AMP*) *in lungs*

Each rat was anaesthetized with pentobarbitone sodium, the abdomen was widely opened and the aorta and inferior vena cava were cut across to exsanguinate the rat. The anterior wall of the thorax was cut away and the lungs were rapidly removed and weighed. The inner hilar regions of each lung were resected and discarded. The remaining peripheral portions of each lung were added to 2 ml ice-cold 0·3 mol/l perchloric acid (PCA) in a pre-weighed glass pot and weighed. In the cold room the lung was finely minced with scissors, homogenized in a blender at 0°C and then centrifuged at 2500 *g* for 7 min. The supernatant was removed and 1 mol/l $KH_2PO_4$ was added to the supernatant to give a final concentration of 0·2 mol/l $KH_2PO_4$, and then the pH was adjusted to 5·5 by adding 5 mol/l KOH. After recentrifugation (2500 *g* for 10 min) the supernatant was removed and used to measure the concentration of c-AMP using a chemical test kit supplied by Boehringer Mannheim (Biochemica) for the radio-isotope dilution protein binding procedure described by Gilman (1970). Before the assay of c-AMP, most supernatants were stored at −70°C for 24–48 h. The pellet obtained from the PCA extract of lung homogenate was resuspended in 5% PCA and the desoxyribose nucleic acid (DNA) concentration was determined by the method of Schmidt and Thannhauser (1945) modified by Munro and Fleck (1966) as previously described (van den Brenk and Stone, 1972). The c-AMP concentration in lung tissue was calculated in terms of pmol c-AMP/mg DNA for individual rats. Two experiments were performed to determine the effect of local x-irradiation of the lungs on c-AMP levels: in the one experiment both lungs were irradiated in each rat and c-AMP concentrations compared with those in sham irradiated controls. In the other experiment, either the right or left lung was locally irradiated in equal numbers of rats in the group and c-AMP and DNA were measured in the irradiated and shielded lung tissue of each rat, after removing and discarding the post-caval lobe situated in the midline of the thorax. The c-AMP values for the 2 sides were compared to minimize any indirect (abscopal) effects of irradiation on concentrations of the nucleotide in lung tissue.

### *Specific activity of DNA in lung*

This was measured in unirradiated and irradiated rats which were killed 2 h after i.p. injection with 50 $\mu$Ci $^3$H-thymidine. The rats were exsanguinated, the lungs were removed and weighed and the DNA concentration and its specific activity in the peripheral lung tissues were measured as described previously (van den Brenk *et al.*, 1975).

### RESULTS

### *Stimulation of tumour CFE in lung by β-adrenergic agonists*

A single i.p. injection of $10^{-5}$–$10^{-4}$ mmol adrenaline or isoprenaline (ISOP)/g body weight caused marked increases in CFE in the lungs when the adrenergic agent was injected from 2 h before to 2 h after i.v. injection of the tumour cells (Table I). The β-adrenergic drug, protokylol, stimulated CFE also. Tumour CFE is high in weanling rats and decreases rapidly with increase in age (van den Brenk, Sharpington and Orton, 1973*b*). Beta-adrenergic drugs stimulated tumour CFE in rats of all ages, the effect increasing with increase in age of host. The rapid decrease in tumour CFE which occurs with increase in age of untreated rats is accompanied by a decrease in tumour growth rate; the tumour colonies become smaller and this contributes to the smaller increases in lung weight produced by the growth of tumour cells in older rats. Besides increasing CFE, adrenaline and ISOP caused the tumour cells to grow to larger-sized colonies, which was mainly responsible for the increases in lung weight produced by the agents in older rats.

Maximum tolerated single doses of a variety of agonists which differed in their pharmacological actions and tissue specificities which were injected 30 min after i.v. injection of tumour cells had no significant effect on tumour CFE. Examples of negative results obtained with the α-adrenergic agent, noradrenaline, and various other agents are shown in Table I. Negative results (not tabulated) were also obtained with a variety of antihistamines, and the diuretic agents, mannitol, edecrin, $NH_4Cl$, $CaCl_2$ and acetazolamide injected in doses of $10^{-4}$–$10^{-3}$ mmol drug/g body weight. The prosta-

TABLE I.—*Effects on Tumour CFE in the Lungs of a Single i.p. Injection of Adrenergic and Other Agents (dose expressed in mmol/g body weight unless stated otherwise) given 30 min after i.v. Injection of Rats with N* W256 *Tumour Cells. Ratios* R($N_L$) *and* R($w$) *Calculated for Number of Tumour Colonies* ($N_L$) *and Lung Weight* ($w$) *Respectively for each Two Groups of Treated and Control Rats** (6–8 *Rats per Group*) *and Significances* ($s$)† *Calculated (see Materials and Methods)*

| $N$ | Age of rats (weeks) | Dose of agent(s) | R($N_L$) | R($w$) |
|---|---|---|---|---|
| $10^4$ | 5 | $5 \times 10^{-6}$ Adrenaline | 0·82 | 1·04 |
| $2 \times 10^3$ | 4 | $10^{-5}$ Adrenaline | 2·95 (s) | 1·22 (s) |
| $10^4$ | 5 | $10^{-5}$ Adrenaline | 1·30 | 1·18 |
| $10^4$ | 5 | $10^{-5}$ Adrenaline | 2·45 (s) | 1·37 (s) |
| $10^4$ | 4 | $10^{-4}$ Adrenaline | 7·78 (s) | 2·48 (s) |
| $10^4$ | 6 | $10^{-4}$ Adrenaline | 3·11 (s) | 1·93 (s) |
| $2 \times 10^3$ | 4 | $10^{-5}$ Isoprenaline | 1·22 | 1·06 |
| $10^4$ | 8 | $5 \times 10^{-5}$ Isoprenaline | 7·73 (s) | 1·20 (s) |
| $10^4$ | 4 | $10^{-4}$ Isoprenaline | 4·30 (s) | 1·59 (s) |
| $10^4$ | 4 | $10^{-4}$ Isoprenaline | 4·27 (s) | 1·71 (s) |
| $10^4$ | 8 | $10^{-4}$ Isoprenaline | 12·19 (s) | 1·38 (s) |
| $10^4$ | 10 | $10^{-4}$ Isoprenaline | 16·75 (s) | 1·01 |
| $10^4$ | 10 | $10^{-4}$ Isoprenaline | 8·00 (s) | 1·02 |
| $10^4$ | 6 | $10^{-4}$ Protokylol | 10·52 (s) | 1·20 |
| $2 \times 10^3$ | 4 | $10^{-5}$ L-Noradrenaline | 1·66 | 1·10 |
| $10^4$ | 4 | 50 μg Angiotensin amide | 1·87 | 1·48 |
| $10^4$ | 7 | 0·2 u Vasopressin (IVI) | 1·40 | — |
| $10^4$ | 7 | 0·4 u Vasopressin (IMI) | 1·73 | — |
| $10^4$ | 4 | $10^{-5}$ Atropine | 0·95 | 0·99 |
| $10^4$ | 5 | $10^{-4}$ o-Acetylcholine | 1·16 | 1·08 |
| $10^4$ | 5 | $10^{-4}$ Histamine | 0·95 | 1·13 |
| $10^4$ | 5 | $10^{-4}$ 5-Hydroxytryptamine | 2·23 | 1·42 (s) |
| $10^4$ | 5 | $10^{-4}$ 2-Bromolysergic acid | 0·81 | 1·02 |
| $10^4$ | 5 | 2 mg $kg^{-1}$ Guanethidine sulphate | 0·39 (s) | 0·94 |
| $10^4$ | 5 | 2 mg $kg^{-1}$ Bretylium tosylate | 0·30 (s) | 0·94 |
| $10^4$ | 5 | 10 mg $kg^{-1}$ Cellulose sulphate | 3·22 (s) | 1·52 (s) |
| $10^4$ | 5 | 2 mg $kg^{-1}$ Guanethidine *plus* 10 mg $kg^{-1}$ cellulose sulphate ‡ | 5·00 (s) | 1·79 (s) |
| $10^4$ | 5 | 2 mg $kg^{-1}$ Guanethidine *plus* $10^{-5}$ adrenaline ‡ | 0·50 (s) | 1·00 |
| $10^4$ | 5 | 2 mg $kg^{-1}$ Bretylium *plus* $10^{-5}$ adrenaline ‡ | 1·30 | 1·18 |

* Control rats injected i.p. with 0·2 ml isotonic saline.
(s) Signifies $P < 0·001$ to $< 0·05$.
‡ Guanethidine sulphate and bretylium tosylate injected i.p. 10 min before CS or adrenaline.

glandins PGA, $PGE_1$, $PGE_2$ and $PGF_{2\alpha}$, histamine and bradykinin failed to increase CFE significantly. I.p. injection of $10^{-4}$ mmol 5-hydroxytryptamine/g body weight, which caused marked prostration of rats, stimulated CFE (Table I, see below). Glucagon injected 30 min to 24 h after i.v. injection of the tumour did not affect CFE.

*Effects of aminophylline on CFE*

I.p. injection of rats with single doses of the phosphodiesterase inhibitor, theophylline ethylene diamine (aminophylline), did not stimulate CFE but 2–3 doses of $10^{-4}$ mmol aminophylline/g body weight, injected from 30 min before to 4 h after injection of the tumour cells caused modest but variable increases in CFE (Table II). Stimulation of CFE in rats treated with adrenaline or ISOP was markedly enhanced by additional treatment with aminophylline (Table III, Fig. 1). The combined effect of ISOP and aminophylline of stimulating CFE was confined to a period from a few hours before to 4 to 6 h after i.v. injection of the tumour cells; CFE was not significantly affected

TABLE II.—*Effect of Treatment of Rats with Aminophylline on Clonogenic Growth in the Lungs of i.v. Injected Tumour Cells. One to Three Doses of Aminophylline (mmol/g body weight) were Injected Intraperitoneally* 30 *min before to* 4 *h after i.v. Injection of Groups of* 6–8 *Rats with* $10^4$ W256 *Tumour Cells*

| Age of rats (weeks) | Dose of aminophylline (no. of doses) | Time(s) | $R(N_L)$* |
|---|---|---|---|
| 4 | $10^{-4}$ (1) | −30 min | 1·64 |
| 4 | $10^{-4}$ (1) | +30 min | 3·00 (s) |
| 5 | $10^{-4}$ (1) | −30 min | 0·80 |
| 5 | $10^{-4}$ (1) | +30 min | 1·08 |
| 5 | $10^{-4}$ (1) | +2 h | 1·33 |
| 5 | $10^{-4}$ (1) | +4 h | 1·76 |
| 5 | $10^{-4}$ (2) | −30 min, +2 h | 2·62 (s) |
| 5 | $10^{-4}$ (2) | +2 h, +4 h | 2·26 (s) |
| 5 | $10^{-4}$ (3) | +30 min, +2 h, +4 h | 3·64 (s) |
| 7 | $10^{-4}$ (1) | −30 min | 1·33 |
| 7 | $10^{-4}$ (2) | −30 min, +2 h | 1·37 |
| 7 | $10^{-4}$ (3) | −30 min, +2 h, +4 h | 2·91 (s) |
| 8 | $10^{-5}$ (1) | +30 min | 1·73 |
| 8 | $3·3\times10^{-5}$ (1) | +30 min | 1·77 |
| 8 | $10^{-4}$ (1) | +30 min | 1·96 |
| 10 | $10^{-4}$ (1) | −30 min | 2·25 |
| 10 | $10^{-4}$ (1) | +30 min | 1·75 |

* See Table I.

FIG. 1.—Effect of injecting i.p. 6-week old female rats with isoprenaline 30 min after the i.v. injection of 5 × $10^3$ W256 cells on the production of tumour macrocolonies in the lungs (○———○), and the effect of the additional i.p. injection of $10^{-4}$ mmol aminophylline/g body weight, given simultaneously with ISOP (□———□). Eight rats per point; control group (●) received isotonic saline.

when the rats were treated with the 2 agents 24 h before or 24 h after injection of the tumour cells (Fig. 2). The combined effect of ISOP and aminophylline of stimulating tumour CFE was comparable with that of injecting an excess of lethally irradiated (LI) tumour cells together with living tumour cells ("Révész Effect") but was not as effective as that obtained when the lungs of rats were locally irradiated (LTI) with 1000 rad 7 days before the i.v. injection of tumour cells (Table III). Combined treatment of rats with ISOP and aminophylline significantly enhanced the effects of LI cells and LTI respectively of stimulating tumour CFE (Table III). A single i.p. injection of $10^{-4}$ mmol aminophylline/g body weight, given 30 min after i.v. injection of the tumour cells, slightly increased the effects of LI cells and LTI of stimulating CFE in young (3–4-week old) but not in older animals (results not tabulated).

*Effects of systemic stress on tumour CFE*

Severe seizures induced in rats by an i.p. injection of pentylenetetrazole (PTZ) given 5–10 min after i.v. injection of W256 cells significantly increased tumour CFE; this effect was abolished when the rats had been anaesthetized to prevent convulsions (Table IV). It is noteworthy that anaesthesia did not similarly affect stimulation of tumour CFE by the injection of $\beta$-adrenergic agents and aminophylline

FIG. 2.—Effect of $10^{-4}$ mmol isoprenaline combined with $10^{-4}$ mmol aminophylline/g body weight, injected together i.p. at various intervals before or after i.v. injection of 5-week old male rats with $10^4$ W256 cells, on the number of tumour macrocolonies produced in the lungs. Six to 8 rats per point: ISOP plus aminophylline (○), controls (●).

(Table V), nor did anaesthesia reduce stimulation of CFE by LTI or LI cells or by inflammatory agents such as cellulose sulphate and Compound 48/80 (results not tabulated). These findings suggested that stimulation of CFE by the chemical convulsant in the rat was an indirect effect due to systemic stress, and that it was probably mediated by the release of adrenal hormones. This conclusion was supported by our finding that physically induced systemic stress ("tumbling" of rats and restraint) similarly stimulated tumour CFE in rats, and that bilateral total ($TA_X$) or medullary ($MA_X$) adrenalectomy abolished this effect of physical stress of stimulating CFE (Table VI). Systemic stress did not stimulate CFE if the rats were stressed 24 h before or 24 h after the i.v. injection of tumour cells. Since $MA_X$ was as effective as $TA_X$ in inhibiting tumour growth in stressed rats, we conclude that the acute release of endogenous adrenaline from the adrenals in states of stress plays a major role in stimulating tumour growth. It is noteworthy that adrenalectomy consistently caused modest but not statistically significant reductions in tumour CFE in unstressed rats and also slightly reduced the effects of LTI on CFE (Table VI, van den Brenk *et al.*, 1973). However, $TA_X$ and $MA_X$ did not significantly reduce stimulation of tumour growth by topical stressors, namely LTI, inflammatory agents or the injection of a $\beta$-adrenergic agent (results not tabulated).

*5-Hydroxytryptamine* (*5OH-T*)

In intact rats tumour CFE was stimulated by large doses of 5OH-T, a chemical mediator of inflammation, but not in rats in which $MA_X$ had been previously per-

TABLE III.—*Effect of Treatment of Rats with Isoprenaline (ISOP) combined with Aminophylline on CFE of i.v. Injected* W256 *Tumour Cells* (6 *Rats used in each Test and corresponding Control Group). The two Compounds were Injected Intraperitoneally as Single Doses* 1 *h after i.v. Injection of Tumour Cells in A and C; in B, the two agents were Injected* 30 *min before, and the same Treatment Repeated* 4 *h after i.v. Injection of Tumour Cells*

| $N$ | Age of rats (weeks) | Dose of agent(s) | R($N_L$) | $P$ | R($w_L$) | $P$ |
|---|---|---|---|---|---|---|
| A | | | | | | |
| $10^4$ | 8 | $10^{-5}$ ISOP + $10^{-5}$ aminophylline | 9·62 | <0·01 | 1·24 | <0·05 |
| $10^4$ | 4 | $10^{-4}$ ISOP + $10^{-4}$ aminophylline | 9·05 | <0·001 | 3·58 | <0·001 |
| $10^4$ | 4 | $10^{-4}$ ISOP + $10^{-4}$ aminophylline | 5·70 | <0·001 | 1·87 | <0·01 |
| $10^3$ | 7 | $10^{-4}$ ISOP + $10^{-4}$ aminophylline | 19·33 | <0·001 | 1·10 | n.s. |
| $10^4$ | 8 | $10^{-4}$ ISOP + $10^{-4}$ aminophylline | 16·65 | <0·001 | 1·67 | <0·01 |
| $10^4$ | 10 | $10^{-4}$ ISOP + $10^{-4}$ aminophylline | 19·25 | <0·001 | 1·07 | n.s. |
| B | | | | | | |
| $10^4$ | 6 | (a) $10^{-4}$ ISOP + $10^{-4}$ aminophylline | 16·60 | <0·001 | 2·01 | <0·01 |
| | 6 | (b) ($2 \times 10^6$ LI cells added only) | 15·50 | <0·001 | 1·43 | <0·01 |
| | 6 | (c) ($2 \times 10^6$ LI cells added; $10^{-4}$ ISOP + $10^{-4}$ aminophylline) | 38·24 | <0·001 | 2·54 | <0·001 |
| | | Difference (b) and (c) | | <0·001 | | <0·001 |
| C | | | | | | |
| $10^4$ | 7 | (a) 1000 rad LTI (Day −7) | 47·00 | <0·001 | 1·34 | <0·001 |
| $10^4$ | 7 | (b) $10^{-4}$ ISOP, $10^{-4}$ aminophylline, 1000 rad LTI (Day −7) | 79·00 | <0·001 | 1·37 | <0·001 |
| | | Difference (a) and (b) | | <0·05 | | n.s. |

In B: $2 \times 10^6$ W256 cells, which had been lethally irradiated (10 K rad x-rays) *in vitro* were added to the i.v. injected $10^4$ intact W256 cells in (b) and (c).

In C: the lungs were locally irradiated with a single dose 1000 rad x-rays 7 days before i.v. injection of the rats with $10^4$ W256 cells in both (a) and (b); the drugs were injected 1 h after the i.v. injection of cells in (b) only.

formed (Tables I, VI). Consequently, stimulation of CFE by 5OH-T is largely attributed to an indirect stressor effect (adrenergic stimulation) of the drug. Similarly, drug induced stress may account for the modest increases in tumour CFE which were produced by other pharmacologically active drugs such as angiotensin amide and vasopressin (Table I) and $PGE_1$, when large doses were given which caused acute prostration. An experiment was also performed in which rats were maintained either at 4°C or at 37°C for 8 h after i. v. injection of tumour cells to induce a mild but more prolonged form of stress, but the treatments did not significantly affect tumour CFE (results not tabulated).

*Sympathetiocolytic agents*

Injection of rats with guanethidine sulphate or bretylium tosylate caused significant reductions in tumour CFE; these agents inhibited the effect of adrenaline of stimulating CFE also, but not that of the inflammatory agent, cellulose sulphate (Table I). Bretylium tosylate did not reduce stimulation of tumour CFE by LTI (results not tabulated).

*α- and β-Adrenergic blocking drugs*

Several experiments were performed to measure the effects of drugs which block α- and β-adrenergic receptors on tumour CFE in the lungs of rats. Results of one of these experiments in 4-week old rats are shown in Table VII, in which dibenyline (α-blocker) and propranolol (β-blocker) were used singly or in combination with the β-agonists, adrenaline and ISOP, respectively. Injected in large doses, dibenyline and propranolol stimulated CFE but did not significantly decrease stimulation of CFE by β-agonists. Experiments in 6- to 10-week old rats gave more variable but similar results,

TABLE IV.—*Effect of Intraperitoneally Injecting Unanaesthetized and Anaesthetized* 6-*week old Female Rats with Pentylene Tetrazole* (*PTZ*)* *on the Number of Lung Tumour Colonies* ($N_L$) *Produced by Intravenously Injecting* $10^3$ W256 *Tumour Cells* (*Groups* 1–7) *or* $10^4$ W256 *Cells* (*Groups* 8, 9). *Eight Rats per Group*

| Group (treatment) | $N_L$ |
|---|---|
| 1. (Saline) | 10 ± 2 |
| 2. (Anaesthetic) | 6 ± 2 |
| 3. (1·2 mg PTZ) | 11 ± 4 |
| 4. (2·5 mg PTZ) | 9 ± 3 |
| 5. (5 mg PTZ) | 40 ± 13 ($P < 0·01$) |
| 6. (Anaesthetic *plus* 5 mg PTZ) | 6 ± 2 |
| 7. (Anaesthetic *plus* 12·5 mg PTZ) | 20 ± 7 ($P < 0·02$) |
| 8. (Saline) | 189 ± 41 |
| 9. (5 mg PTZ) | 299 ± 25 ($P < 0·01$) |

* Rats were injected with PTZ 5–10 min *after* i.v. injection of tumour cells. Rats in Groups 2, 6 and 7 were anaesthetized with 38 mg pentobarbitone sodium/kg body weight injected intraperitoneally 15 min before the tumour cells. The dose of 5 mg PTZ produced one death from convulsions in Groups 5 and 9; the other 7 rats in Group 5 also convulsed. Doses of 1·25 and 2·5 mg PTZ caused no convulsions or deaths; 5 mg PTZ wakened most anaesthetized rats within 10 min without causing convulsions; 12·5 mg PTZ wakened anaesthetized rats within a few min, 3 rats convulsed severely, but all 8 rats in the group recovered. This dose (12·5 mg PTZ) always caused death in unanaesthetized rats from convulsions within 5 min.

TABLE V.—*Effect of Anaesthesia* on Stimulation of Tumour CFE caused by the i.p. Injection of* 4-*week old Female Rats with* $10^{-5}$ *mmol Adrenaline or* $10^{-4}$ *mmol ISOP* plus $10^{-4}$ *mmol Aminophylline/g body weight* 30 *min after i.v. Injection of* $10^4$ W256 *Cells;* 6 *Rats per Group*

| Treatment (anaesthetized ±) | No. of lung colonies (±s.e.) | Lung weight (g) (±s.e.) |
|---|---|---|
| Nil (−) | 81 ± 19 | 1·07 ± 0·03 |
| Nil (+) | 139 ± 33 | 1·17 ± 0·07 |
| $10^{-5}$ Adrenaline (−) | 630 ± 73 | 2·66 ± 0·42 |
| $10^{-5}$ Adrenaline (+) | 550 ± 68 | 2·24 ± 0·33 |
| $10^{-4}$ ISOP *plus* $10^{-4}$ aminophylline (−) | 733 ± 37 | 3·83 ± 0·23 |
| $10^{-4}$ ISOP *plus* $10^{-4}$ aminophylline (+) | 667 ± 40 | 2·86 ± 0·32 |

* 38 mg pentobarbitone sodium IPI 15 min before i.v. injection of tumour.

that is to say, the β-blockers, propranolol and practolol, caused modest increases in CFE and caused slight but not significant reductions in CFE in rats treated with β-agonists.

*Sodium fluoride, imidazole, cyclic AMP*

I.p. injection of rats with single doses of $10^{-5}$ mmol NaF/g body weight caused a significant reduction in tumour CFE, ($R(N_L) = 0·32$), but had no significant effect on stimulation of CFE by ISOP. In these experiments, NaF and ISOP, injected singly or together, did not affect body growth rates, which averaged 4·9–5·2 g/day from the day of injection of drugs and tumour cells till the rats were killed to count tumour colonies 8 days later. I.p. injection of rats with 2 doses of $10^{-4}$ mmol imidazole/g body weight 10 min before and 3 h after i.v. injection of rats with W256 cells had no significant effect on tumour CFE. Incubation of W256 cells with $10^{-4}$ mol/l imidazole for 1 h at 37 °C *in vitro*, as previously described (van den Brenk *et al.*, 1974), did not significantly affect CFE, nor did incubation of W256 cells with $10^{-5}$ mol/l dibutyryl cyclic AMP affect CFE. I.v. injection of rats with $10^{-6}$ mmol dibutyryl cyclic AMP or $10^{-5}$ mmol c-AMP/g body weight 30 min after i.v. injection of W256 cells did not affect tumour CFE (results not tabulated).

*Effects of age, local x-irradiation and β-adrenergic agonists on cyclic AMP in lungs*

The concentration of c-AMP in lung tissue of intact rats decreased with age; in 3 groups of 5–7 female rats killed at 4, 7 and 10 weeks of age the mean concentrations were 77 ± 4, 60 ± 10 and 33 ± 3 pmol c-AMP/mg DNA respectively. Local x-irradiation of the thorax in 10-week old rats with a single dose of 1000 rad caused an initial rapid decrease in c-AMP in lung; this was followed by recovery to values which were about 30% in excess of normal at 3–7 days,

TABLE VI.—*Effects of Physical Stress *and Injection of* $10^{-4}$ *mmol 5-hydroxytryptamine/g Body Weight on Tumour CFE in Intact and Adrenalectomized Rats Injected Intravenously with* $10^4$ W256 *Cells* (6–8 *Rats per Group*). *Mean Gain in Body Weight of Rats* ($\Delta Wg$) *Shown for the* 8 *Days Elapsing from the Day of Injection of Tumour Cells until the Day of Sacrifice; $TA_x$, Bilateral Total Adrenalectomy, $MA_x$, Bilateral Medullary Adrenalectomy*

| | Stress | Adrenalectomy ($TA_x$, $MA_x$) | ΔW(g) (±s.e.) | $N_L$ (±s.e.) | |
|---|---|---|---|---|---|
| A. | Nil | — | 49±2·2 | 106±32 | |
| (5-week old rats) | Stressed 24 h *before* IVI cells | — | 48±0·6 | 97±28 | |
| | Stressed 30 min *after* IVI cells | — | 47±1·0 | 255±55 (s) | |
| | Stressed 24 h *after* IVI cells | — | 46±2·0 | 90±2·0 | |
| B. | Nil | — | 25±2 | 2±0·8 | } (s) |
| (7-week old rats) | Stressed *after* IVI cells | — | 24±2 | 53±12 | } (s) |
| | Nil | $TA_x$ | 8±2 | 0·3±0·3 | } |
| | Stressed *after* IVI cells | $TA_x$ | 9±2 | 2±1·7 | } |
| | Nil | $MA_x$ | 29±1 | 2±1·8 | } |
| | Stressed *after* IVI cells | $MA_x$ | 24±1 | 4±2·5 | } |
| C. | Nil | — | 33±3 | 17±8 | } (s) |
| (6-week old rats) | $10^{-4}$ 5-hydroxytryptamine | — | 40±4 | 165±63 | } (s) } (s) |
| | $10^{-4}$ 5-hydroxytryptamine | $MA_x$ | 38±3 | 11±4 | } (s) |

* In A, groups of 6 rats were stressed by " tumbling " in a drum; in B, the rats were stressed by " restraint " (see Materials and Methods); in C, 5-hydroxytryptamine was injected i.p. 1 h after i.v. injection of the tumour. $TA_x$ and $MA_x$ were performed 24 h before injection of the tumour cells. After $TA_x$, maintanance therapy with cortisone was delayed for 24 h after i.v. injection of tumour cells.

TABLE VII.—*Effect of* $10^{-4}$ *mmol Dibenlyine and* $10^{-4}$ *mmol Propranolol* $g^{-1}$ *Injected Intraperitoneally* 15 *min after i.v. Injection of* 4-*week old Female Rats with* $10^4$W256 *Cells on the number* ($N_L$) *of Lung Tumour Macrocolonies counted* 8 *Days later, and of the Effect of Pretreatment with these Agents on Stimulation of CFE by* $10^{-5}$ *mmol Adrenaline or Isoprenaline* $g^{-1}$ *injected* 30 *min after i.v. Injection of the Tumour Cells** (6 *Rats per Group*)

| | Treatment | $N_L$ | Lung weight (g) |
|---|---|---|---|
| I | Isotonic saline | 81±19 | 1·07±0·03 |
| II | $10^{-5}$ adrenaline | 630±73 | 2·66±0·42 |
| III | $10^{-4}$ dibenyline | 450±106 | 2·19±0·44 |
| IV | $10^{-4}$ propranolol | 186±32 | 1·26±0·42 |
| V | $10^{-4}$ propranolol<br>$10^{-5}$ adrenaline | 199±65 | 1·58±0·38 |
| VI | $10^{-4}$ dibenyline<br>$10^{-5}$ adrenaline | 356±76 | 1·98±0·54 |
| VII | $10^{-4}$ dibenyline<br>$10^{-4}$ propranolol<br>$10^{-5}$ adrenaline | 424±111 | 1·85±0·39 |
| VIII | $10^{-5}$ isoprenaline | 402±66 | 1·87±0·30 |
| IX | $10^{-4}$ propranolol<br>$10^{-5}$ isoprenaline | 298±49 | 1·71±0·39 |

* Adrenergic blockers (propranolol and dibenyline) injected singly or in combination 15 min after i.v. tumour cells, followed by adrenaline or isoprenaline 15 min later, in Groups V, VI, VII and IX.

TABLE VIII.—*Cyclic AMP Concentrations in Lung Produced* 30 *min after an Intraperitoneal Injection of* $10^{-5}$ *mmol Isoprenaline* plus $10^{-4}$ *mmol Aminophylline* $g^{-1}$ *Body Weight in Unirradiated Rats and in Rats given* 1000 *rad Local Thoracic Irradiation* (*LTI*) 7 *Days Previously*

| Treatment | pmol c-AMP/mg DNA I | II |
|---|---|---|
| A. Nil | 60±10 | 64±11 |
| B. LTI | 191±23 | 114±32 |
| C. ISOP, aminophylline | 844±55 | 1167±327 |
| D. LTI, ISOP, aminophylline | 320±44 | 861±58 |

In experiment I, both lungs were irradiated in Groups B and D; in II, the right or left hemithorax was irradiated only in B and D, and c-AMP levels for the corresponding contralateral unirradiated (shielded) lungs in these rats are shown in A and C (5–6 seven-week old female rats per group).

and a return to normal 10 days after LTI (Fig. 3). In 7-week old rats LTI caused more marked increases in c-AMP 7 days after lung irradiation (Table VIII). This effect was confined to the irradiated portions of the lungs. The cause of changes produced by irradiation in c-AMP levels in lung is not known, but the pattern of

FIG. 3.—Cyclic AMP concentrations in the lungs of 10-week old rats given a single dose of 1000 rad x-rays to the thorax (LTI), killed from immediately (0 days) to 10 days after irradiation (5—6 rats per point).

initial decrease followed by recovery to increased levels at 3–7 days, and thereafter a return to normal values, resembles the pattern previously obtained for stimulation of tumour CFE in the lungs of rats by LTI (van den Brenk *et al.*, 1973*a*). A compensatory hyperplastic reaction occurs in the lung after 1000 rad LTI; this causes a marked rise in the specific activity of the DNA which peaks at about 7 days (Fig. 4). The 2 sets of data in Fig. 3 and 4 indicate that reactive increases in cell proliferative activity and c-AMP synthesis, which are induced in the lung by x-ray damage, follow a rather similar time-course and can occur concurrently. A single dose of $10^{-4}$ mmol ISOP caused 14- to 19-fold increases in c-AMP in the lungs of rats killed 15 min after injection of the agonist. In irradiated lungs the $\beta$-agonist was less effective in this respect.

### DISCUSSION

The majority of i.v. injected allogeneic or syngeneic tumour cells or tumour cell aggregates which seed in the lungs and other tissues of rats and mice fail to survive, replicate and produce tumour colonies (Williams and Till, 1966; Hill and Bush, 1969; Withers and Milas, 1973; Brown, 1973; van den Brenk *et al.* 1973*b*; Thompson, 1974). By injecting rats with W256 tumour cells which have been labelled *in vitro* with 5-($^{125}$I)iodo-2′-deoxyuridine or with tritiated thymidine, it has been shown that the majority of tumour cells trap in the lungs but disappear rapidly within the first 6–12 h after injection (van den Brenk *et al.*, 1975). The proportion of the injected tumour cells which survive and produce lung colonies is much higher in 3– to 4-week old weanling rats than in older recipients (van den Brenk *et al.* 1973*b*), but this developmental decrease in tumour CFE with age can be counteracted by inflammatory reactions induced in the tumour bed in the lungs by x-ray or other injury (van den Brenk *et al.*, 1973*a*, 1974). Earlier studies (Fisher and Fisher, 1959*a*, *b*, 1960, 1962; Robinson and Hoppe, 1962; Alexander and Altmeier, 1964) had shown that growth of tumour cells transplanted in the liver and other tissues of the rat was increased by damage to the target tissues caused by contusion or the injection of nitrogen mustard; also, laparotomy stimulated tumour growth. In these experiments it was uncertain whether the effect of injury was topical and directly due to damage to the target tissues, or indirect and caused by systemic stress. Nevertheless, these various observations strongly suggest that tissues of organs which are physiologically intact exhibit considerable

FIG. 4.—Changes in wet weight total DNA and specific activity of DNA of lungs of rats given 1000 rad local irradiation of thorax (LTI) at 4 weeks of age (closed symbols); first measurements (time, 0 days) were made in rats given $^3$H-thymidine immediately after irradiation and killed 2 h later. Open symbols, which show values obtained for unirradiated rats over the same age span as irradiated rats on day of sacrifice, have been plotted on the same time scale.

natural resistance to the survival and growth of a primary challenge of transplanted cancer cells. Such resistance increases with age of host, and in view of the rapidity of its manifestation appears to be *passive* rather than *active* in nature. It cannot be reasonably attributed to the mounting of a specific immunological reaction to the tumour by the host, even if the transplanted cells are allogeneic and immunogenic. Furthermore, in the rat this form of innate resistance acts independently of immunity since CFE of i.v. injected allogeneic tumour cells can be increased by local irradiation of the lungs in an immunized host (van den Brenk *et al.*, 1973*a*), and since irradiation of mice very markedly increased survival and growth of transplanted tumour cells which were essentially non-immunogenic (Hewitt, Blake and Porter, 1973).

In this paper we have shown that innate resistance of host tissues to tumour growth can be greatly reduced by strong stimulation of $\beta$-adrenergic reactions: directly, by injection of rats with large doses of $\beta$-adrenergic agonists or indirectly, by the liberation of endogenous adrenomedullary adrenaline in a state of systemic stress induced in differing ways. Tumour CFE was not similarly affected by maximum tolerated doses of a variety of drugs which interact with other types of receptors and cause different pharmacological effects (Table I), nor by various endogenous mediators of inflammation (van den Brenk *et al.*, 1974). It is significant that whereas lung tissue is richly endowed with $\beta$-adrenergic receptors, it lacks receptors to most of the other agonists which did not stimulate tumour growth in the lungs. An allosteric interaction between an agonist and its corresponding receptor, bound to adenyl cyclase (AC) usually causes an increase in the concentration of the intracellular "second messenger", adenosine-3′-5'-monophosphate cyclic (c-AMP), which is synthesized when AC is activated in the presence of complement (Sutherland and Rall, 1957; Robinson *et al.*, 1971). Cyclic AMP is cleaved to AMP-5′ by intracellular phosphodiesterase (PD); consequently, theophylline and its derivatives which inhibit PD increase the effects of $\beta$-agonists on tissue c-AMP. Adrenaline and ISOP markedly increase c-AMP concentrations in the lungs (Table VIII); prostaglandin $E_1$ causes a more modest increase in lung c-AMP (Jost and Rickenberg, 1971). During postnatal development the c-AMP concentration in various tissues decreases, whereas PD

activity increases (Forn *et al.*, 1970); c-AMP in the lung of rats similarly increases with age (see above). We have been impressed by the correlation between the rise in tumour CFE and the increase in c-AMP concentration which occurs in lungs of rats following local x-irradiation and $\beta$-adrenergic stimulation, as well as by the potentiation of these changes by treatment of rats with a PD inhibitor, and by the corresponding effects of age and inflammatory agents on these parameters. Perturbations of c-AMP metabolism accompany inflammatory reactions in tissues (Bourne *et al.*, 1974) which are growth stimulating (Menkin, 1961). An inflammatory response induced in the lungs of mice by a bacterial infection has been shown to cause a corresponding rise in c-AMP in these tissues, which seems to involve a malfunction at the $\beta$-adrenergic receptor level (Klein, Cory and Fisher, 1974). Stimulation of $\beta$-adrenergic receptors in salivary tissues in rats by ISOP causes marked rises in the salivary c-AMP concentration (Malamud, 1969) and induces rapid mitogenesis and sialadenotrophism which is due to proliferative, combined with hypertrophic, growth of salivary acinar cells (Selye, Veilleux and Cantin, 1961). These correlations between c-AMP levels and stimulation of growth of normal tissues *in vivo* caused us to speculate that agents which increase the c-AMP concentrations in a target organ also stimulate survival and growth of seeded tumour cells, and that changes in innate resistance of tissues to tumour growth *in vivo* are basically caused by perturbations in cyclic nucleotide metabolism.

We have postulated previously that stimulation of tumour CFE by inflammation of the tumour bed is due to the local release of trophic hormone(s), (growth stimulating substances, GSS), which are concerned in stimulating replicative growth of the normal tissues in the repair process associated with inflammation, and likewise that of seeded neoplastic cells (van den Brenk and Sharpington, 1971; van den Brenk *et al.*, 1973*a*). The secretion of most hormones is a uni-directional event in which c-AMP functions as the second intracellular messenger (Robinson *et al.*, 1971). Perturbations of cell metabolism caused in states of topical and systemic stress, in which $\beta$-adrenergic receptors are stimulated and c-AMP synthesis increased, are conceivably associated with trophic reactions of a protective reparative nature, mediated by secretion of GSS. The sialadenotrophic effect of ISOP in rats provides an example of the growth promoting actions of $\beta$-adrenergic stimulation. In the case of the polypeptide fibroblast growth factor (FGF), the growth stimulating effect on normal tissue fibroblasts *in vitro* (Gospodarowicz, 1974) has been shown to be due to the stimulation of guanosine -3′-5′-monophosphate cyclic (c-GMP) synthesis in "resting" ($G_0$) cells. This nucleotide, with hydrocortisone and $Ca^{++}$ as co-factors, is self-sufficient in acting as a mitogenic trigger which programmes fibroblasts for DNA synthesis and replication in the absence of serum (Rudland, Gospodarowicz and Seifert, 1974). Similarly, various other mitogenic substances which programme cells for replicative growth stimulate synthesis of c-GMP as a prelude to DNA synthesis (Abell and Monahan, 1973). *In vitro*, GSS present in serum also stimulates growth of neoplastic cells. The autonomous growth pattern of neoplastic (transformed) cells appears to be associated with an enzymatic imbalance which endows the cell with a capacity to generate its own growth signal. Preparing tumour cells in suspension for transplantation *in vivo* and their bathing in blood and tissue fluids would tend to remove GSS originally present in their micro-environment. This would make the supply of GSS by normal tissues at the site of seeding important in stimulating growth of the tumour cells before they become established and are able to condition their own microenvironment with GSS. Evidence of the importance of adequate concentrations of GSS for stimulating survival and clonogenic growth of normal and neoplastic mammalian cells is provided not only by serum requirements *in vitro* but by the use of conditioned media and "feeder layers" (Puck and Marcus, 1956), and *in vivo* by the effect of lethally irradiated (LI) cells (Révész, 1958), phenomena which depend on the elaboration and release of GSS by metabolically active cells.

Although increases in c-GMP precede replicative cell growth, increases in intracellular c-AMP *in vitro* and *in vivo* generally occur when replicative growth ceases spontaneously or is inhibited (Abell and Monahan, 1973) and so-called pleiotypic effects are induced, consisting of decreases in the rates of RNA, protein and DNA synthesis and a stimulation of protein degradation (Kolata, 1973). These changes affect both bi-directionally and unidirectionally controlled physiological systems (Goldberg *et al.*, 1972) stimulate functions such as contraction, storage of cellular products and secretion, and cause a modulation of cellular activity from a state of growth towards that of contact-inhibition and cyto-differentiation. Thus, in skin c-AMP levels fluctuate with a pronounced diurnal rhythm, reaching a maximum during the resting phase of growth (Marks and Grimm, 1972). *In vitro*, c-AMP, dibutyryl cyclic AMP and ISOP also inhibit DNA synthesis in PHA- and concanavalin A stimulated lymphocytes (Abell, Kamp and Johnson, 1970; Johnson and Abell, 1970; Krug *et al.*, 1972) and the growth of HeLa and L cells (Ryan and Heidrick, 1968). Otten, Johnson and Pastan (1971) studied fibroblasts in logarithmic growth phase and growth of virus transformed 3T3 cells *in vitro;* they found that intracellular levels of c-AMP were inversely proportional to DNA synthesis. The studies of Averner, Brock and Jost (1972) have indicated that c-AMP directly inhibits transcription and not replication of DNA. Under certain circumstances only does c-AMP appear to stimulate DNA synthesis and division; this occurs in cells that have already been programmed for growth by a promotional, as opposed to a mitogenic, effect (Rixon, Whitfield and MacManus, 1970).

It seems difficult to reconcile our observations that tumour CFE is stimulated in tissues which have been stressed by inflammatory reactions and $\beta$-adrenergic stimulation, which cause tissue c-AMP to increase, with the finding that c-AMP causes pleiotypic effects and initially inhibits growth in cells and tissues. This raises the possibility that "take" and survival of seeded cancer cells in tissues in the initial stages depend not so much on stimulation of mitogenesis as on other physiological changes which enhance adhesion and attachment of the cells to normal tissue (endothelial) surfaces and bring about a better physiological and cyto-architectural integration of the tumour cells with the host, to subserve their metabolic needs for the reprogramming of gene expression. This supposes that a preliminary modulation of tumour cell function occurs in which growth is temporarily held in abeyance—a change which is readily reversible and may be, perhaps wrongly, designated "differentiation" (Weiss, P., 1973) and which would be favoured by increases in tissue c-AMP. The mechanisms responsible for attachment of seeded cancer cells to endothelial and other tissue surfaces *in vivo* have received widespread attention; the roles of haemocoagulation and fibrinolysis in particular have been extensively investigated in this respect. However, a recent comprehensive quantitative study by Röttinger, Sedlacek and Suit (1975) has failed to show that anticoagulant therapy affects tumour growth in either normal or irradiated tissues, and treating mice with the defibrinating agent ancrod did not significantly affect clonogenic growth or spread of subcutaneously implanted tumour cells (Peters and Hewitt, 1974). Increases in the fibrinolytic activity and clotting of blood have been reported to occur after the injection of adrenaline and in stress (Biggs, MacFarlane and Pilling, 1947); evidence of a basic diurnal rhythm of fibrinolytic activity has been obtained also (Fearnley, Balmforth and Fearnley, 1957). Under certain conditions, for obscure reasons, deposition of fibrin and fibrinolysis do appear to affect survival and growth of implanted tumour cells (Peters and Hewitt, 1974) but treatment with anticoagulants does not significantly reduce the effects of x-irradiation and various other stressors of stimulating tumour CFE in the lungs of rats (van den Brenk *et al.*, 1973*a*). Recent studies have shown that early interactions between tumour and host, which lead to attachment and the provision of adhesion gradients for directional migration (haptotaxis; Carter, 1967), invasion and clonogenic growth of cancer cells in tissues depends on chemically mediated cell-to-cell signals which modulate interactions between surface proteins. Cyclic nucleotides appear to act in this way as chemical signals which govern contact inhibition and confluency of growth and so-called "differentiation". Intracellular c-AMP concentrations have been found to increase with confluency and contact inhibition has been attributed to activation of membrane AC (Heidrick and Ryan, 1970, 1971). The adhesion of Ehrlich ascites tumour cells to a plastic surface was not affected by c-AMP; this finding was attributed to the presence of PD in the serum used since PD resistant dibutyryl c-AMP, applied as a continouus signal, decreased adhesion (Weiss, L., 1973). More recently, evidence has been obtained that c-AMP, applied intermittently to cells in culture as pulses, provides a chemotactic signal whereby the cells communicate during aggregation (Shaffer, 1975; Gross, 1975), and that their exposure to a short pulse of c-AMP causes them in turn to secrete c-AMP. This cell–cell reaction induces physiological modulation of a cell population from a state of active multiplicative growth to one of "aggregation competence". We postulate that the attachment of a seeded tumour cell to an endothelial surface may be an aggregation phenomenon which similarly requires the generation of a pulsatile c-AMP signal by the tumour bed (endothelium) for the tumour cell to become structurally and physiologically assimilated and nurtured by the sharing of a common micro-environment. Thus, Lettré (1952) showed that ATP caused enhanced flattening and adherence of cells to surfaces, even when the cells were in mitosis. Weiss (1961) has argued that such cellular expansion at an interface is a metabolically "active" rather than passive process. The higher net negative charge on transformed cells than on normal cells (Abercrombie, Heaysman and Karthausen, 1957) also would be expected to facilitate adhesion of the cancer cell to endothelium. Thus, it has been shown that when transformed cells are seeded *in vitro* on a monolayer of untransformed cells in the absence of a plasma coagulant, they do not form the heaped up colonies seen on glass, but spread as a layer over the normal cells (Vogt and Rubin, 1961). Interestingly enough, similar experiments by Stoker (1964) showed that the rate of cell division decreased during this form of spreading and migratory growth of transformed cells on normal cells—a finding which would be expected if this modulation of growth was mediated by a c-AMP signal. Consequently, the enhancement of "take" and replicative growth of tumour cells *in vivo* by stressors may depend on a biphasic mechanism, in which stimulation of c-AMP synthesis in normal tissue in the first instance causes adherence and aggregation competence of the tumour cells and secondarily induces the secretion by normal tissues of GSS which stimulates tumour cell mitogenesis and replication, *i.e.*, a sequence of physiological changes which would appear to be in keeping with a "Yin and Yang" (Goldberg *et al.*, 1972) or "see-saw" (Rudland *et al.*, 1974.) hypothesis, in which c-AMP and c-GMP have antithetical actions in the regulation of growth as well as in other bi-directionally controlled physiological processes.

The two key metabolic pathways which are involved in the cyclic nucleotide "see-saw" and lead to cell synthesis of the two "antagonistic" messengers c-AMP and c-GMP are linked by inosine-5′-monophosphate (IMP)—the nucleotide which occupies a strategic position in purine metabolism; changes in the regulation of IMP synthesis by IMP-dehydrogenase which occur in states of regenerative and neoplastic growth have recently been linked with competitive routes for utilization of IMP in the "expression of degrees of malignancy" (Jackson, Weber and Morris, 1975). A GSS fraction has been prepared from brain which has a biphasic effect on myocytes *in vitro*, comprising induction of proliferative growth accompanied by differentiation (Gospodarowicz, 1975)—"antagonistic" actions in this respect which resemble the actions of the $\beta$-adrenergic agent ISOP in salivary tissues *in vivo* and in which cyclic nucleotide metabolism plays a key role. Beta-adrenergic agents were relatively less effective in stimulating tumour CFE in lung than inflammatory reactions. This may be due to the more prolonged action (chronicity) of the inflammation in causing cyclic nucleotide malfunction than that of injecting rats with a rapidly metabolized $\beta$-adrenergic drug. This is supported by the finding that treatment with the PD inhibitor, aminophylline, which increases and prolongs the effect of $\beta$-adrenergic agents on tissue c-AMP, also increased their effect (and that of other stimulants) on tumour CFE (Table III, Fig. 3).

Tumour CFE was stimulated by both $\alpha$- and $\beta$-adrenergic blocking agents; the latter did not significantly alter the stimulating effects of $\beta$-adrenergic agonists on CFE. These findings do not necessarily conflict with the view that activation of receptor bound AC stimulates tumour growth, since $\beta$-blocking agents stimulate c-AMP activity in certain tissues (Allison, Denman and Barnes, 1971) and do not effectively block some $\beta$-adrenergic receptors (Gillis, Pearle and Hockman, 1974). *In vitro*, solubilized AC can be activated by fluoride (Schramm and Naim, 1970) and imidazole stimulates c-AMP-phosphodiesterase activity in subcellular fractions (Sutherland and Rall, 1958), but neither fluoride nor imidazole appears to be active *in vivo* (Robinson *et al.*, 1971). This would explain their failure to alter tumour CFE in rats treated with $\beta$-adrenergic agonists.

The anti-adrenergic agents, guanethidine and bretylium decreased tumour CFE in rats; they also decreased the effects of adrenaline on CFE but did not significantly decrease stimulation of CFE by inflammatory reactions induced by cellulose sulphate or x-irradiation, which are mediated by the release of non-adrenergic agonists. Depression of tumour CFE by guanethidine and bretylium would appear to be primarily due to the widespread depletion and decreased release of adrenergic transmitter substances stored in the peripheral and ganglionic nerve terminals. This suggests that in states of stress, apart from the release of adrenergic hormones, autonomic nerve stimuli may be directly involved in modulating tissue functions which affect tumour growth.

Cyclic AMP participates in the early events of the immune reaction *in vitro* (Bösing-Schneider and Kolb, 1973). Principally, it prevents spleen lymphocytes differentiating into antibody producing cells. The time scale for the action of c-AMP on immune induction which precedes proliferation of immunocytes is at least 24 h (Bösing-Schneider, 1975). Since the majority of tumour cells which seed in the lungs succumb within the first 24 h, it seems unlikely that the increased c-AMP synthesis in states of stress stimulates tumour growth by suppressing the development of active immunity.

Since it is likely that topical and systemic stress influences the metastatic spread of spontaneous and induced tumours in mammals in the same way as it affects experimentally seeded cancer cells, it follows that psychosomatic factors may influence the natural history of cancer in man to a greater extent than is commonly acknowledged. Also, the possibility arises that a variety of therapeutic agents can affect growth of metastases; in repeated doses, certain cytotoxic agents such as cyclophosphamide increased tumour CFE in rats (unpublished results). Other non-cytotoxic but pharmacologically active agents may do likewise but may be more specific in this respect in singling out those target tissues which are endowed with receptors to the particular agonist. For example, although glucagon did not stimulate tumour CFE (or c-AMP synthesis) in the lungs, the possibility arises that it may do so in the liver, where glucagon causes a c-AMP mediated hyperglycaemic effect. Conversely, a drug which causes stress can indirectly stimulate tumour growth in those tissues which react weakly or not at all to the agonist, but react to adrenaline (*e.g.* stimulation of CFE in the lungs by 5-hydroxytryptamine; Table VI).

### REFERENCES

ABELL, C. W., KAMP, C. W. & JOHNSON, L. D. (1970) Effects of Phytohaemagglutinin and Isoproterenol on DNA Synthesis in Lymphocytes from Normal Donors and Patients with Chronic Lymphocytic Leukaemia. *Cancer Res.*, **30**, 717.

ABELL, C. W. & MONAHAN, T. M. (1973) The Role of Adenosine 3', 5' -Cyclic Monophosphate in the Regulation of Mammalian Cell Division. *J. cell Biol.*, **59**, 549.

ABERCROMBIE, M., HEAYSMAN, J. E. & KARTHAUSEN, H. M. (1957) Social Behaviour of Cells in Tissue Culture. III. Mutual Influence of Sarcoma Cells and Fibroblasts. *Expl cell Res.*, **13**, 276.

ALEXANDER, J. W. & ALTMEIER, W. A. (1964) Susceptibility of Injured Tissues to Haematogenous Metastases: An Experimental Study. *Ann. Surg.*, **159**, 933.

ALLISON, A. C., DENMAN, A. M. & BARNES, R. D. (1971) Cooperating and Controlling Functions of Thymus-derived Lymphocytes in Relation to Autoimmunity. *Lancet*, ii 135.

AVERNER, M. J., BROCK, M. L. & JOST, J. -P. (1972) Stimulation of Ribonucleic Acid Synthesis in Horse Lymphocytes by Exogenous Cyclic Adenosine 3', 5' -Monophosphate. *J. biol. Chem.*, **247**, 413.

BIGGS, R., MACFARLANE, R. G. & PILLING, J. (1947) Observations on Fibrinolysis Experimental Activity Produced by Exercise or Adrenaline. *Lancet*, i, 402.

BÖSING-SCHNEIDER, R. (1975) Differential Effects of Cyclic AMP on the *in vitro* Induction of Antibody Synthesis. *Nature, Lond.*, **256**, 137.

BÖSING-SCHNEIDER, R. & KOLB, M. (1973) Influence of Cyclic AMP on Early Events of the Immune Induction. *Nature, Lond.*, **244**, 224.

BOURNE, H. R., LICHTENSTEIN, L. M., MELMON, K. L., HENNEY, C. S., WEINSTEIN, Y. & SHEARER, G. M. (1974) Modulation of Inflammation and Immunity by Cyclic AMP. *Science, N.Y.*, **184**, 19.

BROWN, J. M. (1973) The Effect of Lung Irradiation on the Incidence of Pulmonary Metastases in Mice. *Br. J. Radiol.*, **46**, 613.

CARTER, S. B. (1967) Haptotaxis and the Mechanism of Cell Motility. *Nature, Lond.*, **213**, 256.

FEARNLEY, G. R., BALMFORTH, G. & FEARNLEY, E. (1957) Evidence of a Diurnal Fibrinolytic Rhythm: With a Simple Method of Measuring Natural Fibrinolysis. *Clin. Sci.*, **16**, 645.

FISHER, B. & FISHER, E. R. (1959*a*) Experimental Studies of Factors Influencing Hepatic Metastases: III. Effect of Surgical Trauma with Special Reference to Liver Injury. *Ann. Surg.*, **150**, 731.

FISHER, B. & FISHER, E. R. (1959*b*) Experimental Evidence in Support of the Dormant Tumor Cell. *Science, N.Y.*, **130**, 918.

FISHER, B. & FISHER, E. R. (1960) Experimental Studies of Factors Influencing Hepatic Metastases: IV. Effect of Cirrhosis. *Cancer, N.Y.*, **13**, 860.

FISHER, B. & FISHER, E. R. (1962) Experimental Studies of Factors Influencing Hepatic Metastases: XI. Effect of Hepatic Trauma in Hypophysectomised Animals. *Proc. Soc. exp. Biol. Med.*, **109**, 62.

FORN, J., SCHONHOFER, P. S., SKIDMORE, I. F. & KRISHNA, G. (1970) Effect of Aging on the Adenyl Cyclase and Phosphodiesterase Activity of Isolated Fat Cells of Rat. *Biochim. biophys. Acta*, **208**, 304.

GILLIS, R. A., PEARLE, D. L. & HOCKMAN, T. (1974) Failure of Beta-adrenergic Blockade to Prevent Arrhythmias Induced by Sympathetic Nerve Stimulation. *Science, N.Y.*, **185**, 70.

GILMAN, A. G. (1970) A Protein Binding Assay for Adenosine-3'-5'-Cyclic Monophosphate. *Proc. natn. Acad. Sci. U.S.A.*, **67**, 305.

GOLDBERG, N. HADDOX, M. K., HARTLE, D. K. & HADDON, J. W. (1972) The Biological Role of Cyclic 3-5-Guanosine Monophosphate. *Proc. 5th Int. Cong. Pharmacol.*, V, 146.

GOSPODAROWICZ, D. (1974) Localisation of a Fibroblast Growth Factor and Its Effect Alone and with Hydrocortisone on 3T3 Cell Growth. *Nature, Lond.*, **249**, 123.

GOSPODAROWICZ, D. (1975) Presence in Brain of a Mitogenic Agent Promoting Proliferation of Myoblasts in Low Density Culture. *Nature, Lond*, **256**, 216.

GROSS, J. D. (1975) Periodic Cyclic AMP Signals and Cell Differentiation. *Nature, Lond.*, **255**, 522.

HEIDRICK, M. L. & RYAN, W. L. (1970) Cyclic Nucleotides on Cell Growth *in vitro*. *Cancer Res.*, **30**, 376.

HEIDRICK, M. L. & RYAN, W. L. (1971) Adenosine 3', 5' -Cyclic Monophosphate and Contact Inhibition. *Cancer Res.*, **31**, 1313.

HEWITT, H. B., BLAKE, E. & PORTER, E. H. (1973) The Effect of Lethally Irradiated Cells on the Transplantability of Murine Tumours. *Br. J. Cancer*, **28**, 123.

HILL, R. P. & BUSH, R. S. (1969) A Lung-colony Assay to Determine Radiosensitivity of the Cells of a Solid Tumour. *Int. J. radiat. Biol.*, **15**, 435.

JACKSON, R. C., WEBER, G. & MORRIS, H. P. (1975) IMP Dehydrogenase, an Enzyme Linked with Proliferation and Malignancy. *Nature, Lond*, **256**, 331.

JOHNSON, L. D. & ABELL, C. W. (1970) The Effects of Isoproterenol and Cyclic Adenosine 3′, 5′ Phosphate on Phytohaemagglutinin-stimulated DNA Synthesis in Lymphocytes Obtained from Patients with Chronic Lymphatic Leukemia. *Cancer Res.*, **30**, 2718.

JOST, J.-P. & RICKENBERG, H. V. (1971) Cyclic AMP. *A. rev. Biochem.*, **40**, 741.

KRUG, U., KRUG, F. & CUATRECASAS, P. (1972) Emergence of Insulin Receptors on Human Lymphocytes During *in vitro* Transformation. *Proc. natn. Acad. Sci. U.S.A.*, **64**, 1349.

KLEIN, T. W., CORY, J. G. & FISHER, C. W. (1974) Binding of cAMP and Phosphodiesterase Activity in Lung and Diaphragm Preparations of Normal and *B. pertussis*-infected Mice. *Fedn Proc.*, **33**, 762.

KOLATA, G. B. (1973) Cyclic GMP: Cell Regulatory Agent? *Science, N.Y.*, **182**, 149.

LETTRÉ, H. (1952) Some Investigations on Cell Behavior Under Various Conditions. *Cancer Res.*, **12**, 847.

MALAMUD, D. (1969) Adenyl Cyclase Activity in Isoproterenol-stimulated Mouse Salivary Glands. *J. cell Biol.*, **43**, 202.

MARKS, F. & GRIMM, W. (1972) Diurnal Fluctuation and β-adrenergic Elevation of Cyclic AMP in Mouse Epidermis *in vivo*. *Nature, New Biol.*, **240**, 178.

MENKIN, V. (1961) Studies of the Growth Promoting Factors of Exudates in Reference: (a) to its Effect on the Development of Spontaneous Neoplasms in Tumour Susceptible Mice; (b) to its Presence in Rabbit Exudate, (c) in its Comparative Growth Potential in Organs of Immature and Adult Animals. *Path. Biol.*, **9**, 861.

MUNRO, H. N. & FLECK, A. (1966) Recent Developments in the Measurement of Nucleic Acids in Biological Materials. *Analyst*, **91**, 78.

OTTEN, J., JOHNSON, G. S. & PASTAN, I. (1971) Cyclic AMP Levels in Fibroblasts: Relationship to Growth Rate and Contact Inhibition of Growth. *Biochem. biophys. Res. Commun.*, **42**, 1198.

PETERS, L. J. (1975) Enhancement of Syngeneic Murine Tumour Transplantability by Whole-body Irradiation: A Non-immunogenic Phenomenon. *Br. J. Cancer*, **31**, 293.

PETERS, L. J. & HEWITT, H. B. (1974) The Influence of Fibrin Formation on the Transplantability of Murine Tumour Cells: Implications for the Mechanism of the Révész Effect. *Br. J. Cancer*, **29**, 279.

PUCK, T. T. & MARCUS, P. I. (1956) Action of X-Rays on Mammalian Cells. *J. exp. Med.*, **103**, 653.

RÉVÉSZ, L. (1958) Effect of Lethally Damaged Tumour Cells Upon the Development of Admixed Viable Cells. *J. natn. Cancer Inst.*, **20**, 1157.

RIXON, R. H., WHITFIELD, J. F. & MACMANUS, J. P. (1970) Stimulation of Mitotic Activity in Rat Bone Marrow and Thymus by Exogenous Adenosine 3′, 5′-Monophosphate (Cyclic AMP). *Expl cell Res.*, **63**, 110.

ROBINSON, G. A., BUTCHER, R. W. & SUTHERLAND, E. W. (1971) *Cyclic AMP*. New York: Academic Press.

ROBINSON, K. P. & HOPPE, E. (1962) The Development of Blood-borne Metastases. Effect of Local Trauma and Ischaemia. *Archs Surg.*, **85**, 720.

RÖTTINGER, E. M., SEDLACEK, R. & SUIT, H. D. (1975) Ineffectiveness of Anticoagulation in Experimental Radiation Therapy. *Eur. J. Cancer*. In the press.

RUDLAND, P. S., GOSPODAROWICZ, D. & SEIFERT, W. (1974) Activation of Guanyl Cyclase and Intracellular Cyclic GMP By Fibroblast Growth Factor. *Nature, Lond.*, **250**, 741.

RYAN, W. L. & HEIDRICK, M. L. (1968) Inhibition of Cell Growth *in vitro* By Adenosine 3′, 5′-Monophosphate. *Science, N.Y.*, **162**, 1484.

SCHMIDT, G. & THANNHAUSER, S. J. (1945) Determination of DNA, RNA and Phosphoproteins in Animal Tissue. *J. biol. Chem.*, **161**, 83.

SCHRAMM, M. & NAIM, E. (1970) Adenyl Cyclase of Rat Parotid Gland. Activation by Fluoride and Norepinephrine. *J. biol. Chem.*, **245**, 3225.

SELYE, H. (1954) Interactions Between Systemic and Local Stress. *Br. med. J.*, i, 4872.

SELYE, H., VEILLEUX, R. & CANTIN, N. (1961) Excessive Stimulation of Salivary Gland Growth by Isoproterenol. *Science, N.Y.*, **133**, 44.

SHAFFER, B. M. (1975) Secretion of Cyclic AMP Induced by Cyclic AMP in the Cellular Slime Mould Dictyostelium Discoideum. *Nature, Lond.*, **255**, 549.

STOKER, M. (1964) Regulation of Growth and Orientation in Hamster Cells Transformed by Polyoma Virus. *Virology*, **24**, 165.

SUTHERLAND, E. W. & RALL, T. W. (1957) The Properties of an Adenine Ribonucleotide Produced with Cellular Particles, ATP, $Mg^{++}$ and Epinephrine or Glucagon. *J. Am. chem. Soc.*, **79**, 3608.

SUTHERLAND, E. W. & RALL, T. W. (1958) Fractionation and Characterization of a Cyclic Adenine Ribonucleotide Formed by Tissue Particles. *J. biol. Chem.*, **232**, 1077.

THOMPSON, S. C. (1974) Pulmonary Tissue Changes and Their Effect on Metastatic Growth of Mouse Tumours. *Ph.D. Thesis*, University of London.

VAN DEN BRENK, H. A. S. & KELLY, H. (1973) Stimulation of Growth of Metastases by Local X-irradiation in Kidney and Liver. *Br. J. Cancer*, **28**, 349.

VAN DEN BRENK, H. A. S. & KELLY, H. (1974) Potentiating Effect of Prior Local Irradiation of the Lungs on Pulmonary Metastases. *Br. J. Radiol.*, **47**, 332.

VAN DEN BRENK, H. A. S. & SHARPINGTON, C. (1971) Effect of Local X-irradiation of a Primary Sarcoma in the Rat on Dissemination and Growth of Metastases: Dose-response Characteristics. *Br. J. Cancer*, **25**, 812.

VAN DEN BRENK, H. A. S. & STONE, M. G. (1972) Effect of X-radiation on Salivary Gland Growth in the Rat. *Int. J. radiat. Biol.*, **21**, 247.

VAN DEN BRENK, H. A. S. BURCH, W. M., ORTON, C. & SHARPINGTON, C. (1973*a*) Stimulation of Clonogenic Growth of Tumour Cells and Metastases in the Lungs by Local X-radiation. *Br. J. Cancer*, **27**, 291.

VAN DEN BRENK, H. A. S., BURCH, W. M., KELLY, H. & ORTON, C. (1975) Venous Diversion Trapping and Growth of Blood-borne Cells *en route* to the Lungs. *Br. J. Cancer*, **31**, 46.

VAN DEN BRENK, H. A. S., SHARPINGTON, C. & ORTON, C. (1973*b*) Macrocolony Assays in the Rat of Allogeneic Y-P388 and W-256 Tumour Cells Injected Intravenously: Dependence of Colony Forming Efficiency on Age of Host and Immunity. *Br. J. Cancer*, **27**, 134.

Van den Brenk, H. A. S., Stone, M., Kelly, H., Orton, C. & Sharpington, C. (1974) Promotion of Growth of Tumour Cells in Acutely Inflamed Tissues. *Br. J. Cancer*, **30**, 246.

Vogt, P. K. & Rubin, H. (1961) Localization of Infectious Virus and Viral Antigen in Chick Fibroblasts During Successive Stages of Infection with Rous Sarcoma Virus. *Virology*, **13**, 528.

Weiss, P. (1961) Guiding Principles in Cell Locomotion and Cell Aggregation. *Expl cell Res. Suppl.*, **8**, 260.

Weiss, L. (1973) Studies on Cellular Adhesion in Tissue Culture. XIIA Some Effects of Prostaglandins and Cyclic Nucleotides. *Expl cell Res.*, **81**, 57.

Weiss, P. (1973) Differentiation and Its Three Facets: Facts, Terms and Meaning. *Differentiation*, **1**, 3.

Williams, J. F. & Till, J. E. (1966) Formation of Lung Colonies by Polyoma-transformed Rat Embryo Cells. *J. natn. Cancer Inst.*, **37**, 177.

Withers, H. R. & Milas, L. (1973) The Influence of Preirradiation of Lung on Development by Artificial Pulmonary Metastases of Fibrosarcoma in Mice. *Cancer Res.*, **33**, 1931.


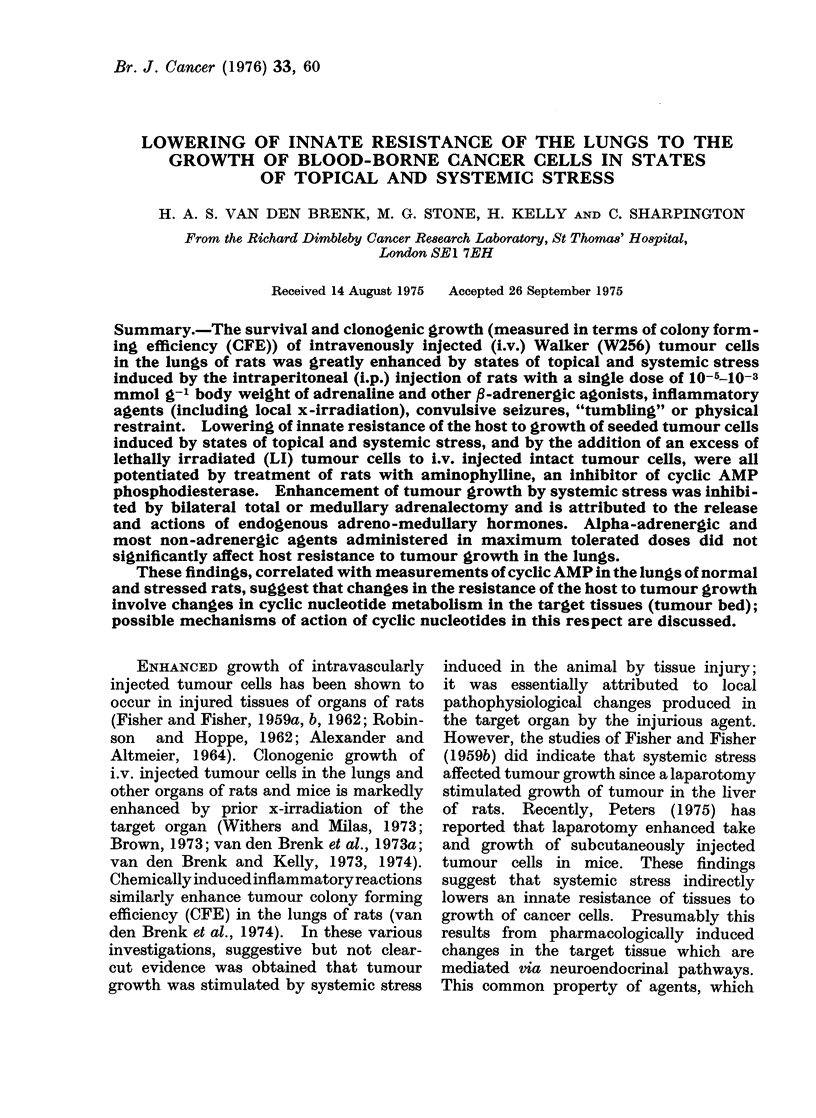

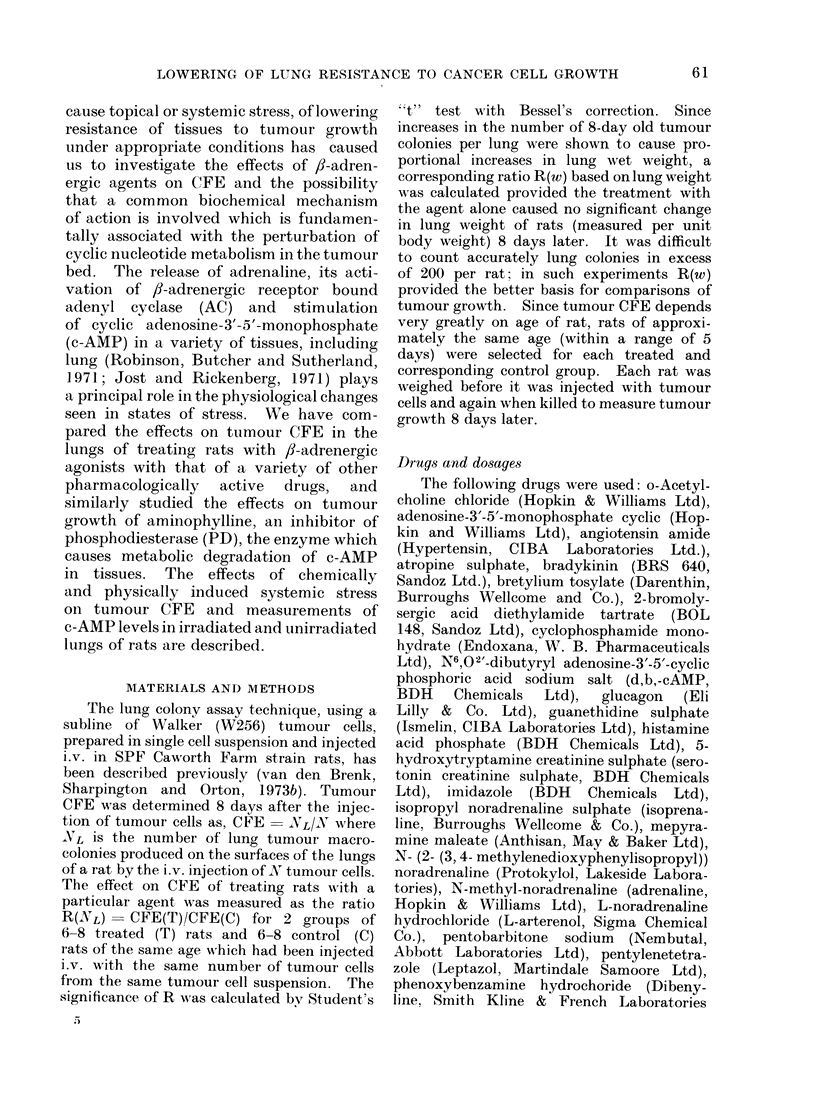

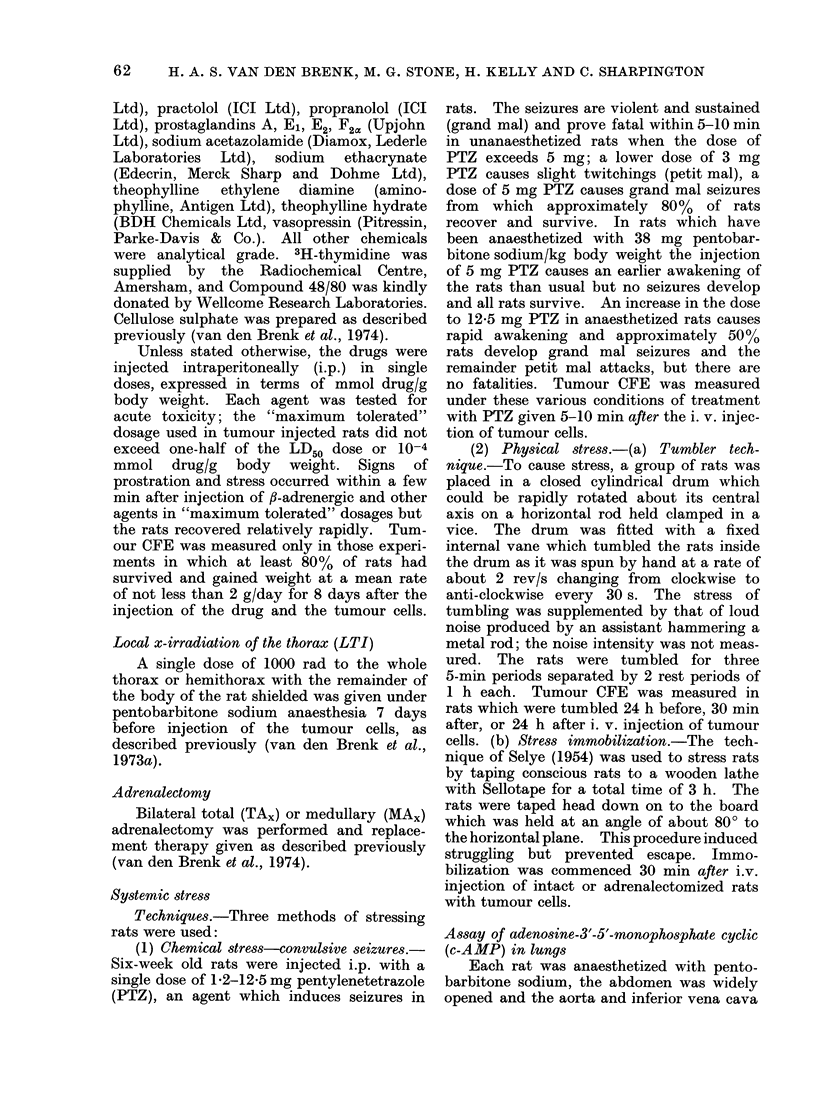

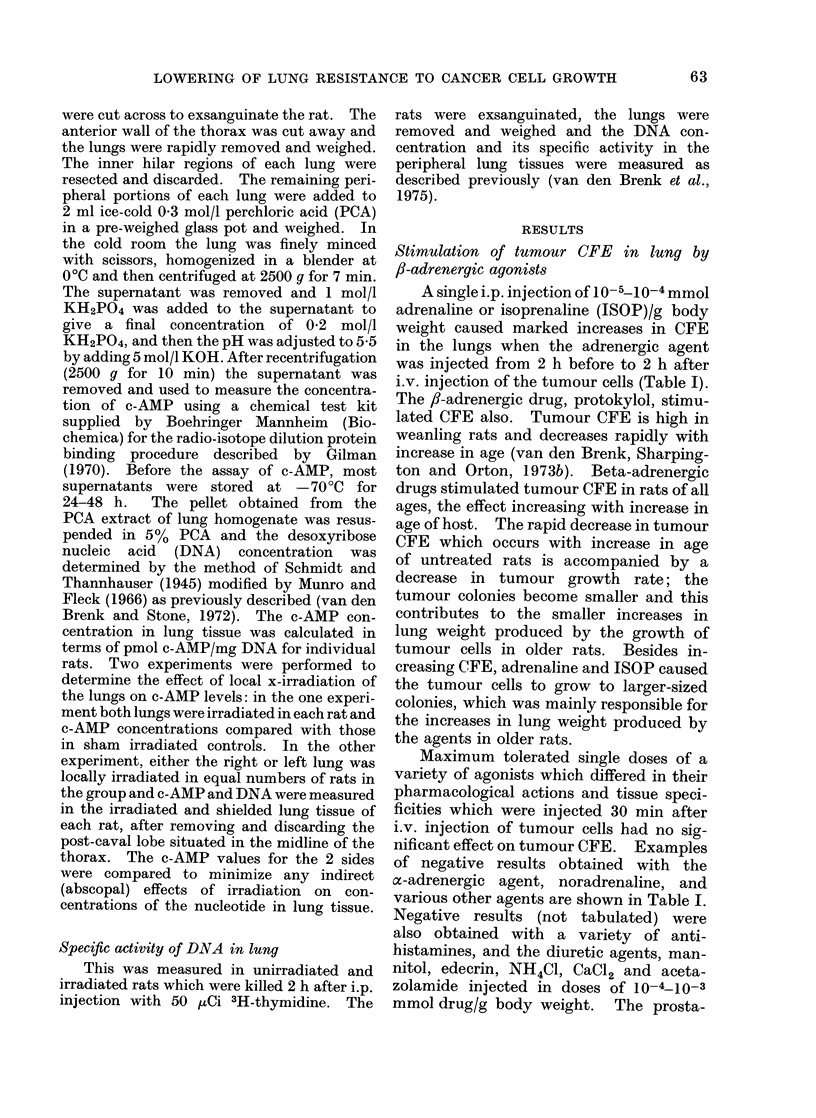

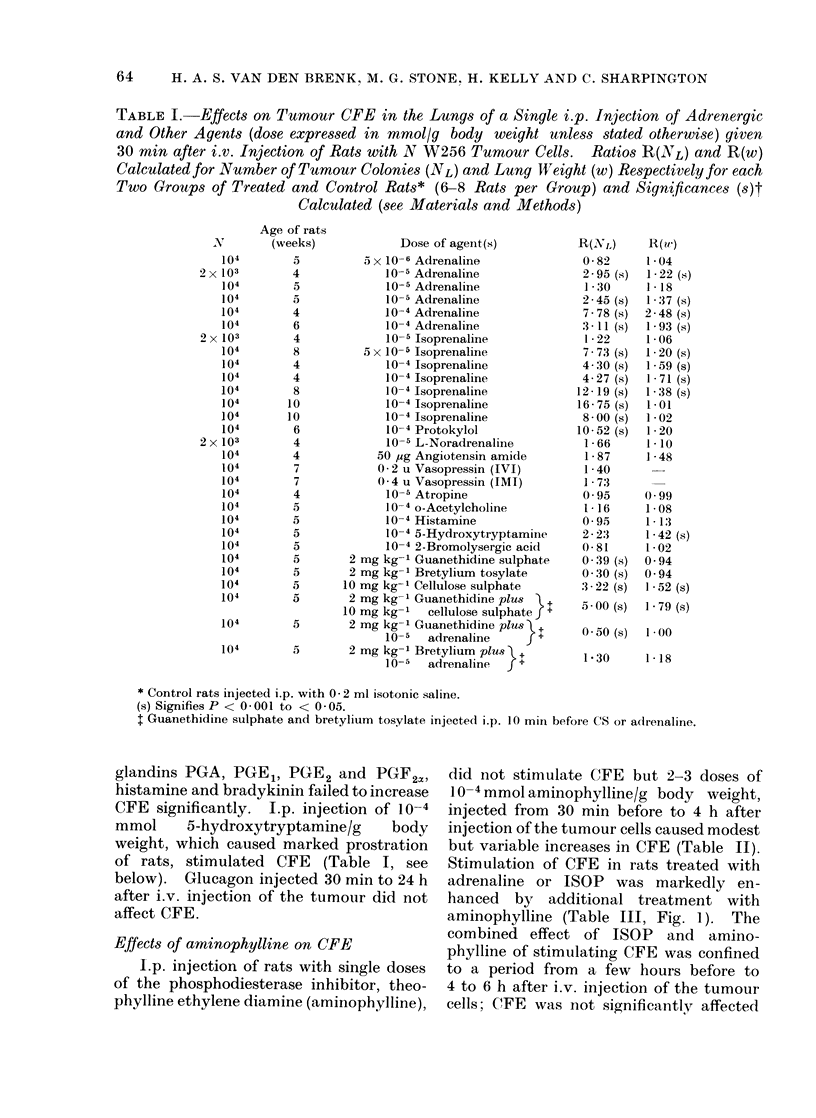

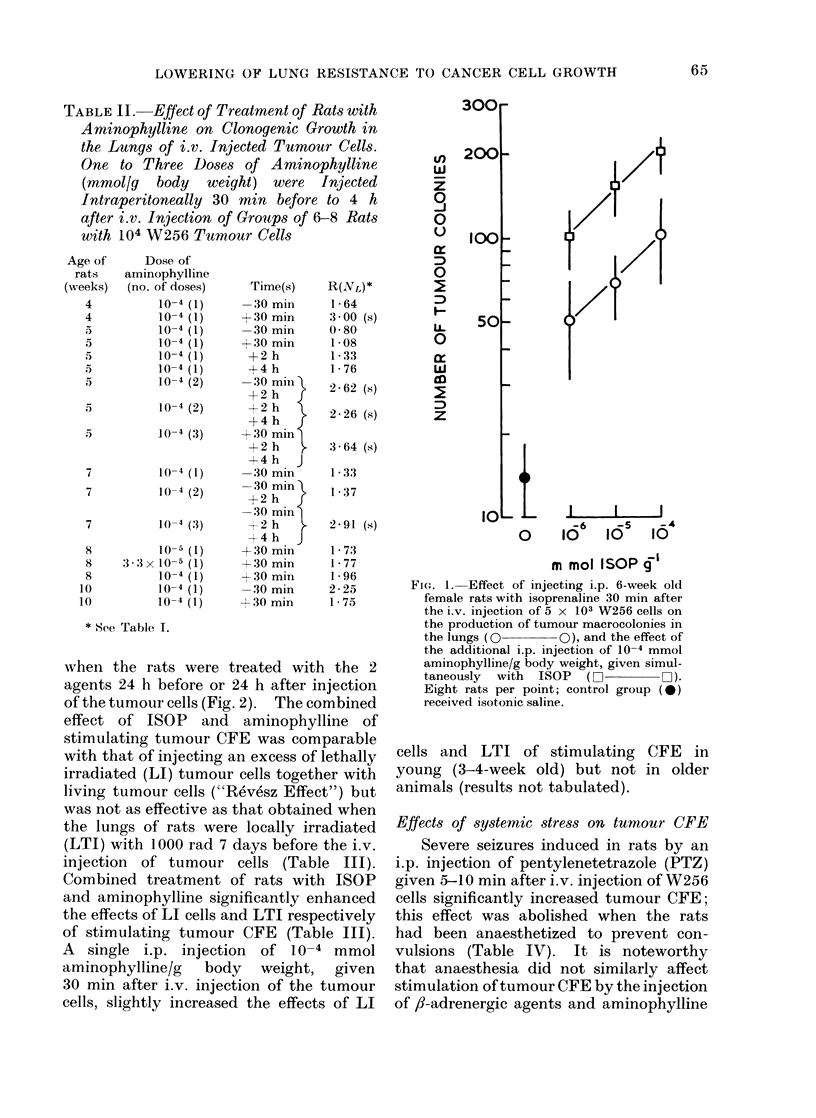

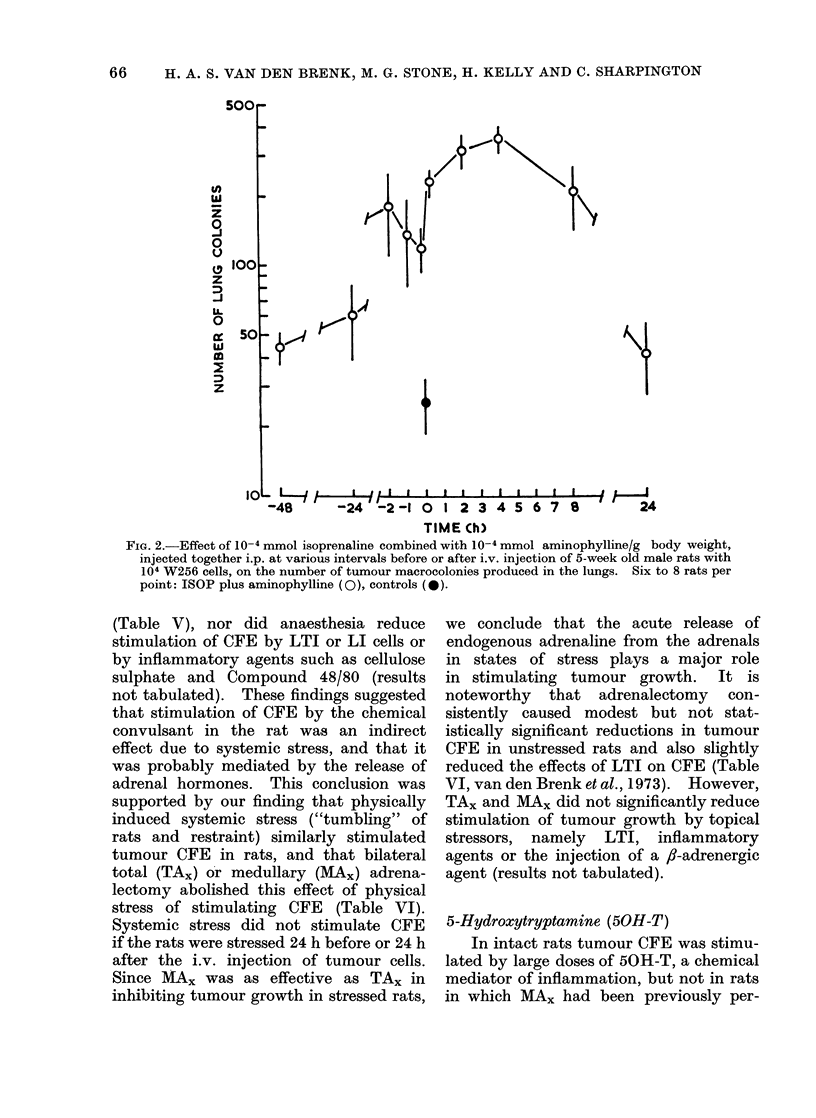

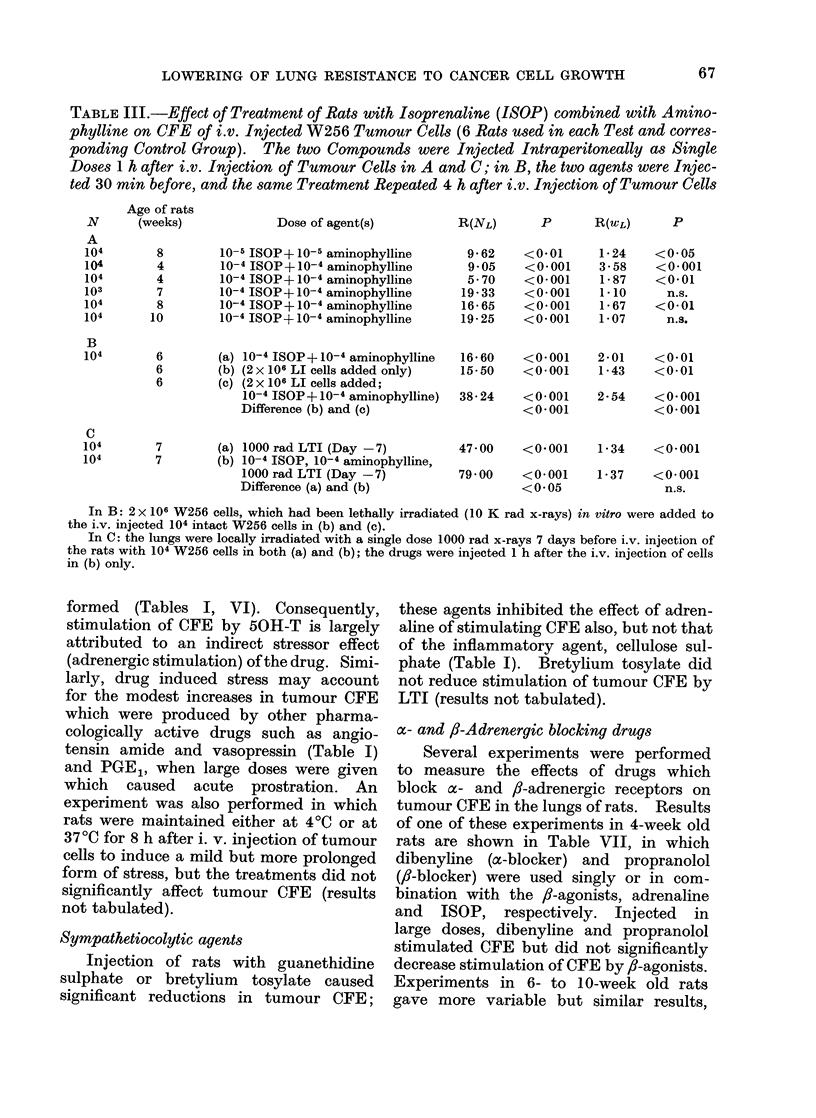

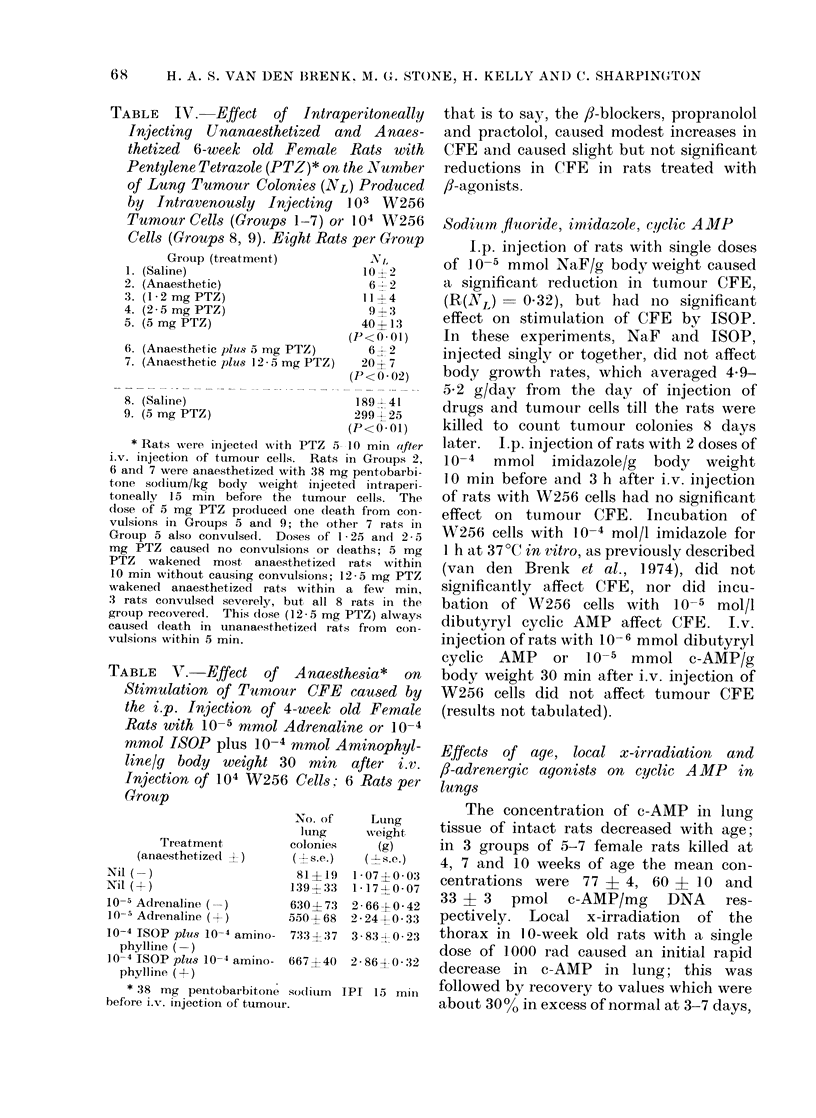

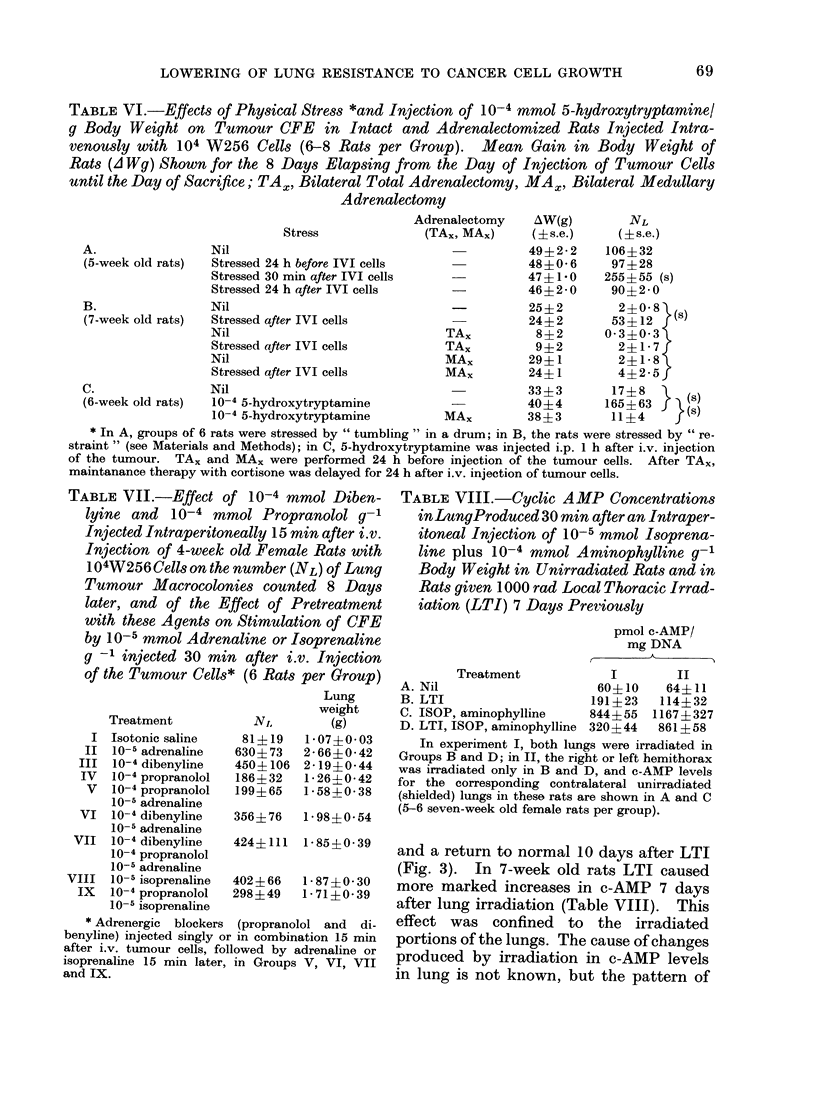

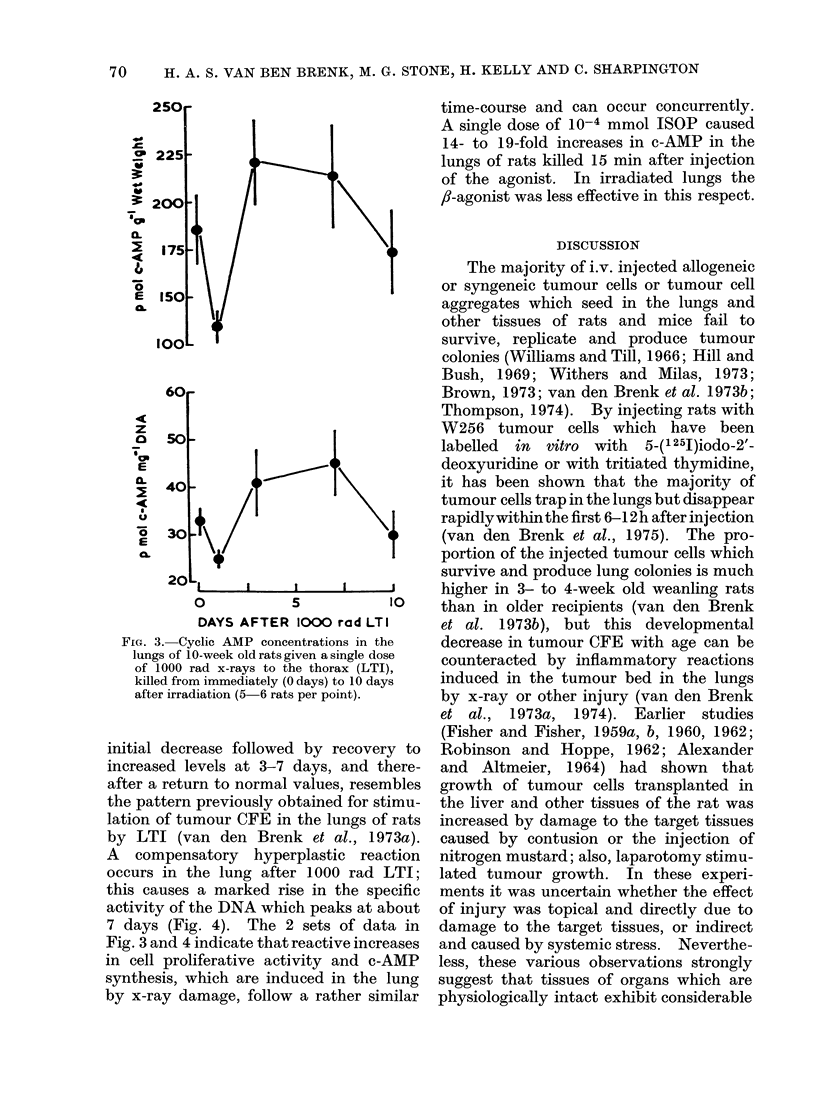

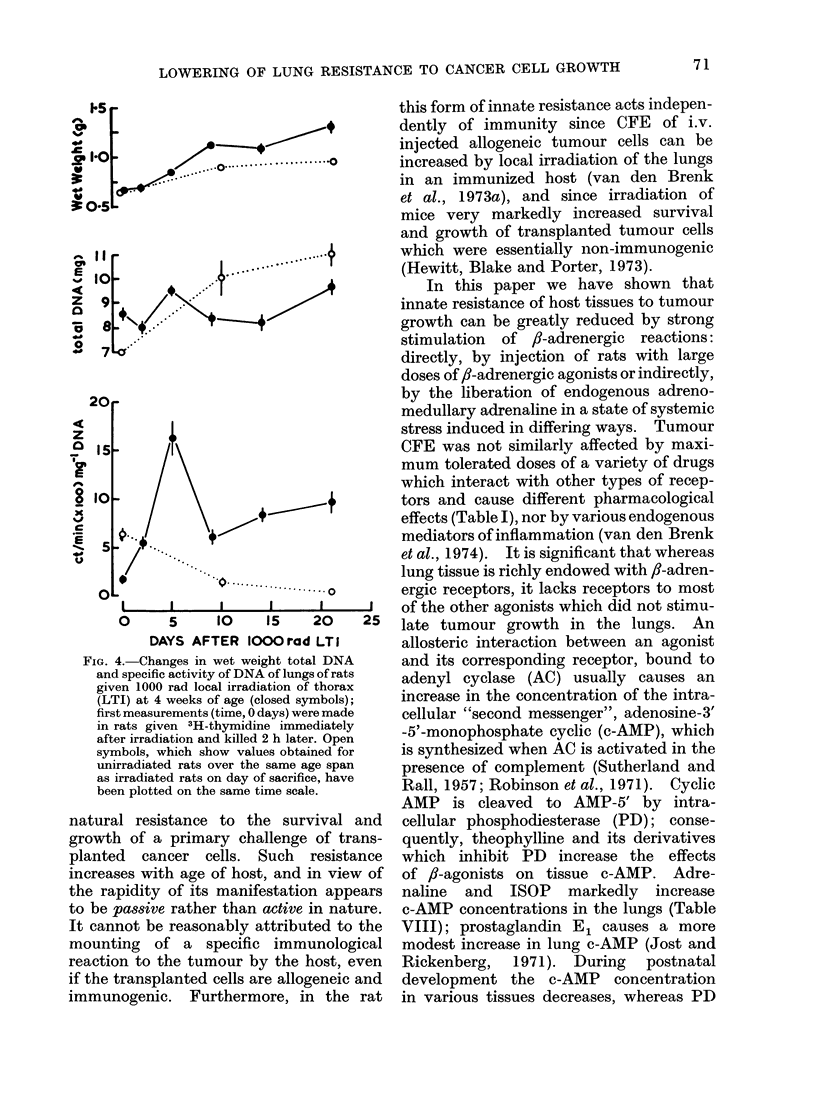

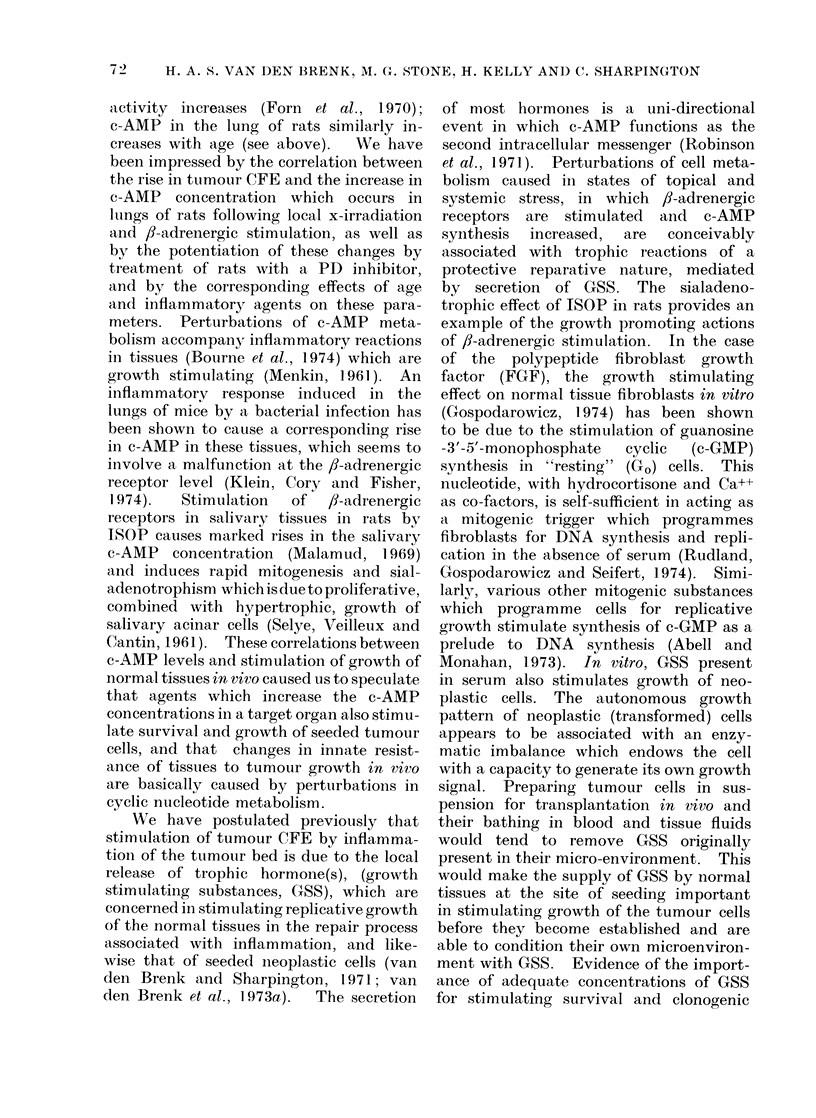

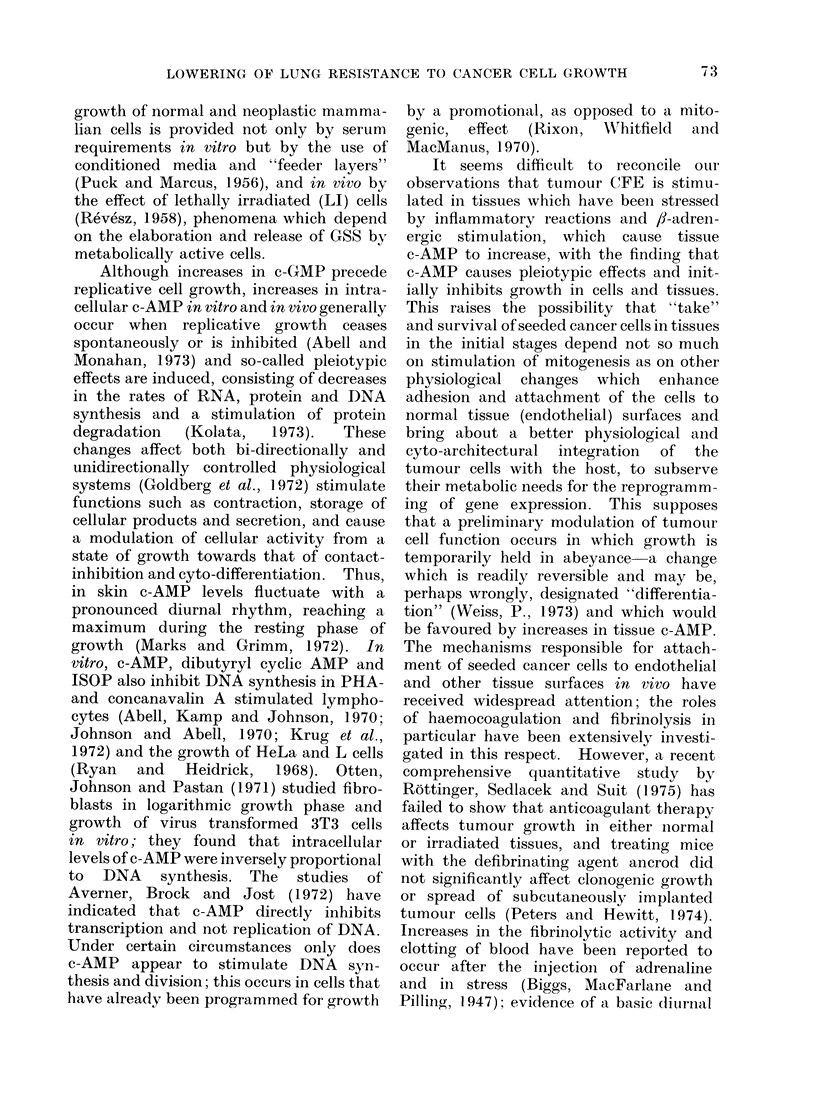

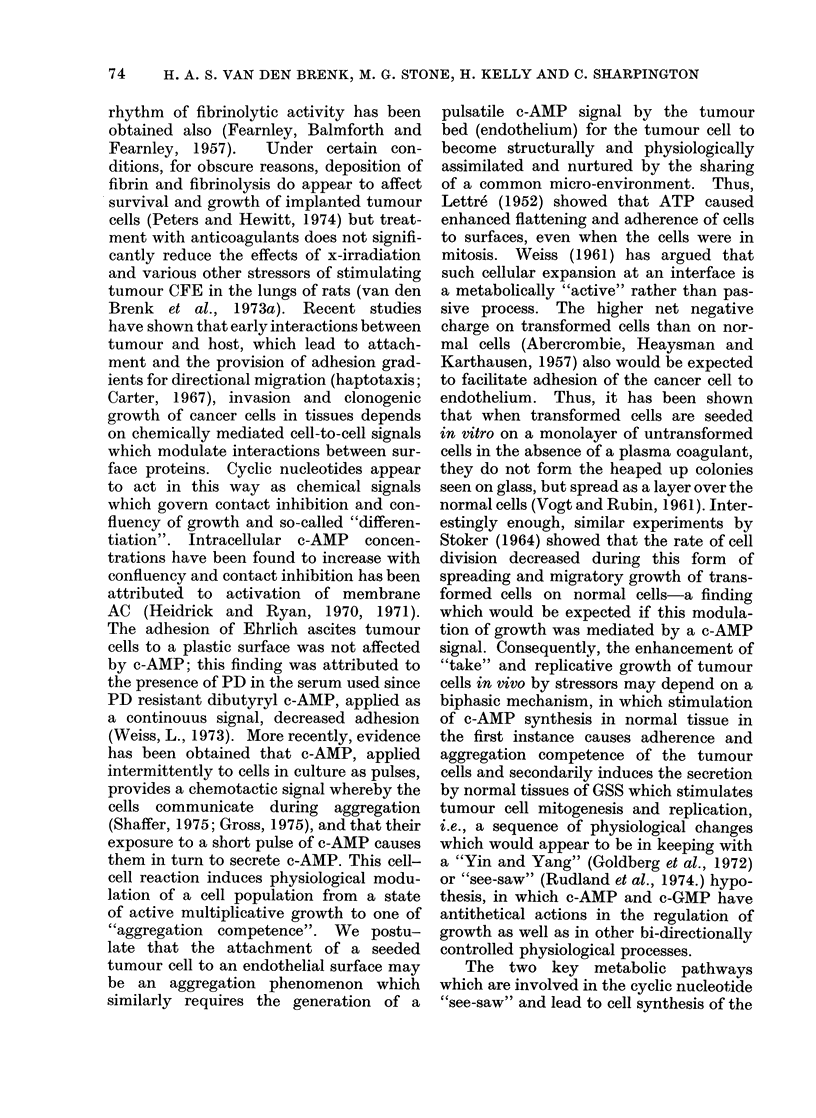

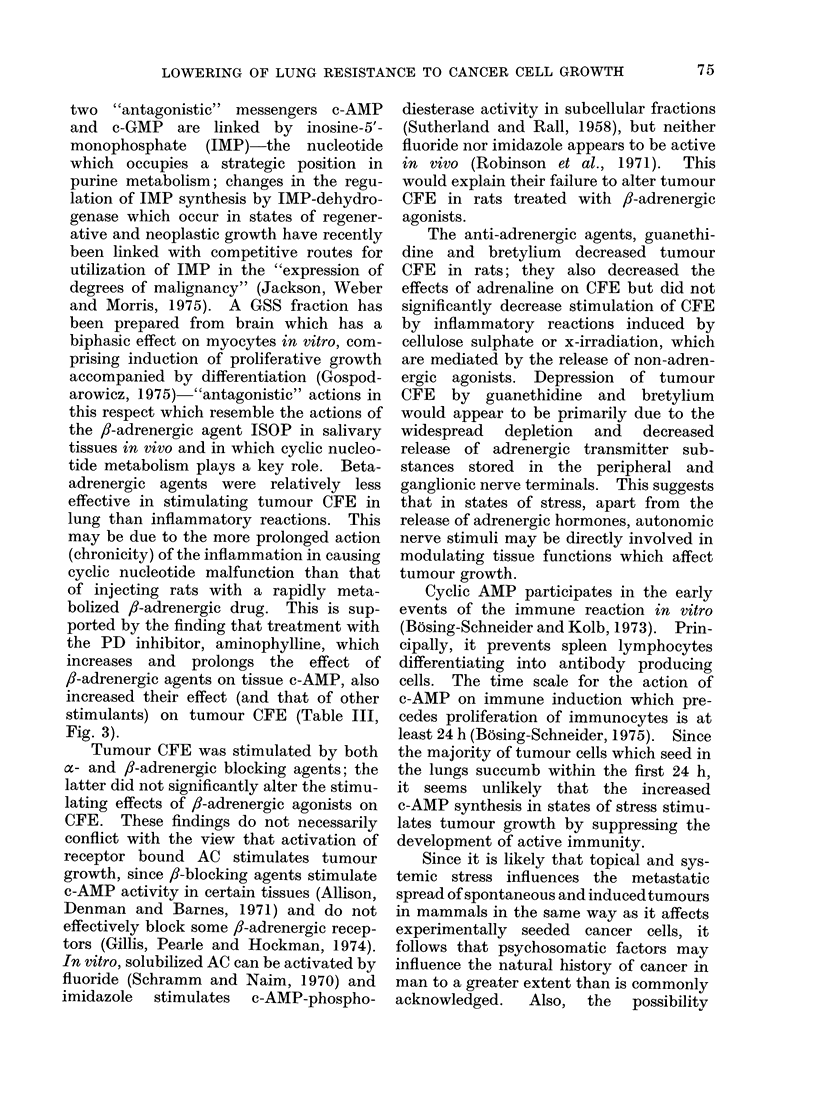

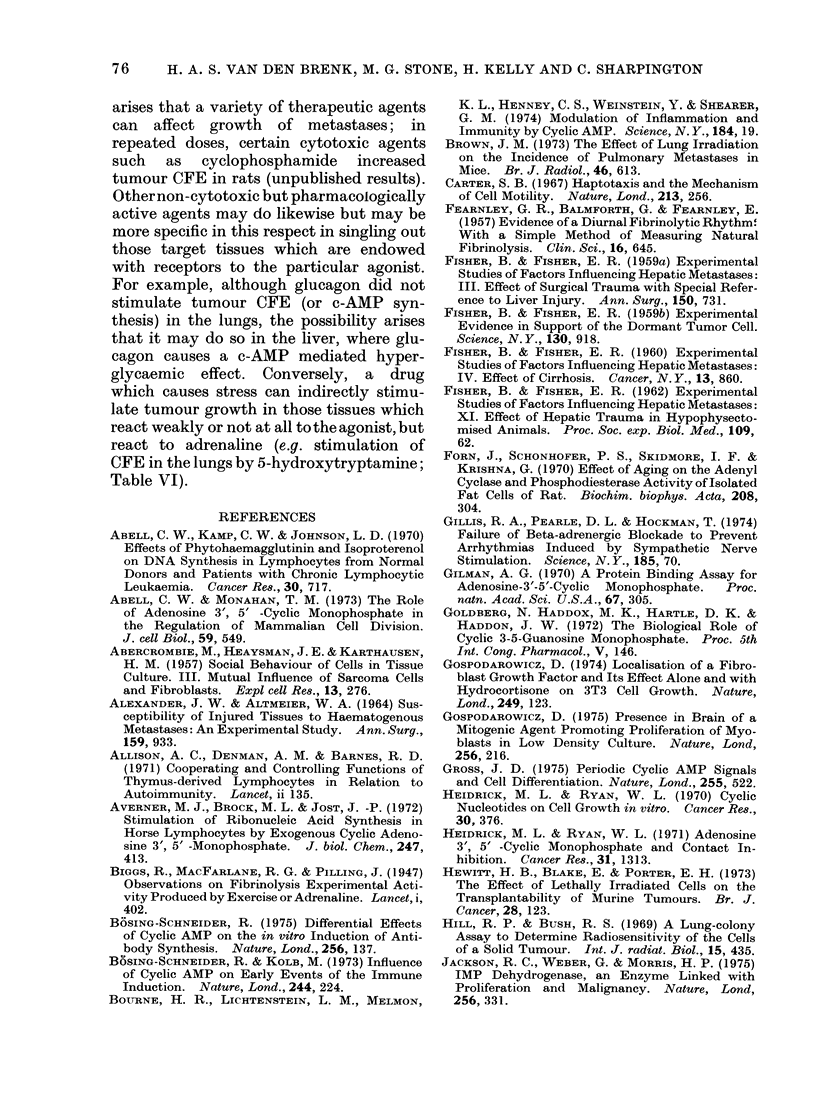

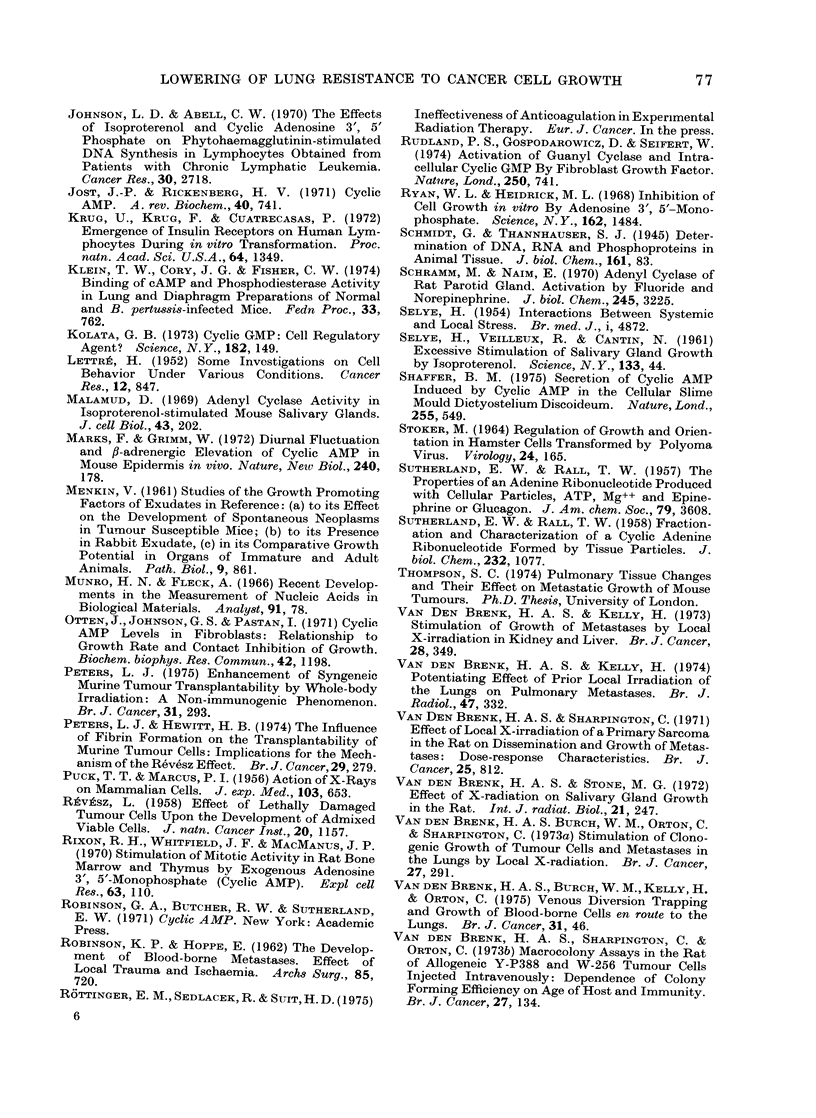

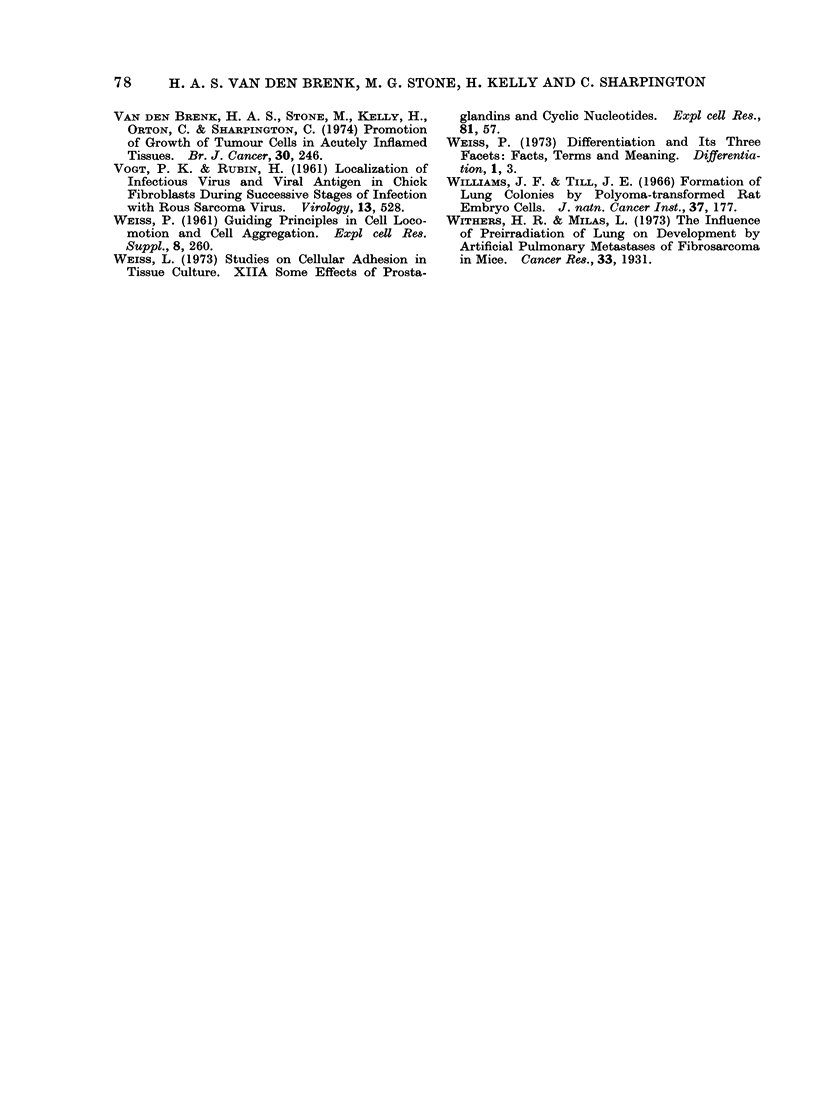


## References

[OCR_02277] ABERCROMBIE M., HEAYSMAN J. E., KARTHAUSER H. M. (1957). Social behaviour of cells in tissue culture. III. Mutual influence of sarcoma cells and fibroblasts.. Exp Cell Res.

[OCR_02283] ALEXANDER J. W., ALTEMEIER W. A. (1964). SUSCEPTIBILITY OF INJURED TISSUES TO HEMATOGENOUS METASTASES: AN EXPERIMENTAL STUDY.. Ann Surg.

[OCR_02264] Abell C. W., Kamp C. W., Johnson L. D. (1970). Effects of phytohemagglutinin and isoproterenol on DNA synthesis in lymphocytes from normal donors and patients with chronic lymphocytic leukemia.. Cancer Res.

[OCR_02271] Abell C. W., Monahan T. M. (1973). The role of adenosine 3',5'-cyclic monophosphate in the regulation of mammalian cell division.. J Cell Biol.

[OCR_02289] Allison A. C., Denman A. M., Barnes R. D. (1971). Cooperating and controlling functions of thymus-derived lymphocytes in relation to autoimmunity.. Lancet.

[OCR_02295] Averner M. J., Brock M. L., Jost J. P. (1972). Stimulation of ribonucleic acid synthesis in horse lymphocyte by exogenous cyclic adenosine 3',5'-monophosphate.. J Biol Chem.

[OCR_02313] Bosing-Schneider R., Kolb M. (1973). Influence of cyclic AMP on early events of the immune induction.. Nature.

[OCR_02318] Bourne H. R., Lichtenstein L. M., Melmon K. L., Henney C. S., Weinstein Y., Shearer G. M. (1974). Modulation of inflammation and immunity by cyclic AMP.. Science.

[OCR_02323] Brown J. M. (1973). The effect of lung irradiation on the incidence of pulmonary metastases in mice.. Br J Radiol.

[OCR_02308] Bösing-Schneider R. (1975). Differential effects of cyclic AMP on the in vitro induction of antibody synthesis.. Nature.

[OCR_02328] Carter S. B. (1967). Haptotaxis and the mechanism of cell motility.. Nature.

[OCR_02332] FEARNLEY G. R., BALMFORTH G., FEARNLEY E. (1957). Evidence of a diurnal fibrinolytic rhythm; with a simple method of measuring natural fibrinolysis.. Clin Sci.

[OCR_02344] FISHER B., FISHER E. R. (1959). Experimental evidence in support of the dormant tumor cell.. Science.

[OCR_02338] FISHER B., FISHER E. R. (1959). Experimental studies of factors influencing hepatic metastases. III. Effect of surgical trauma with special reference to liver injury.. Ann Surg.

[OCR_02354] FISHER B., FISHER E. R. (1962). Experimental studies of factors influencing hepatic metastases. XI. Effect of hepatic trauma in hypophysectomized animals.. Proc Soc Exp Biol Med.

[OCR_02349] FISHER E. R., FISHER B. (1960). Experimental studies of factors influencing hepatic metastases. IV. Effect of cirrhosis.. Cancer.

[OCR_02361] Forn J., Schonhofer P. S., Skidmore I. F., Krishna G. (1970). Effect of aging on the adenyl cyclase and phosphodiesterase activity of isolated fat cells of rat.. Biochim Biophys Acta.

[OCR_02368] Gillis R. A., Pearle D. L., Hoekman T. (1974). Failure of beta-adrenergic receptor blockade to prevent arrhythmias induced by sympathetic nerve stimulation.. Science.

[OCR_02374] Gilman A. G. (1970). A protein binding assay for adenosine 3':5'-cyclic monophosphate.. Proc Natl Acad Sci U S A.

[OCR_02385] Gospodarowicz D. (1974). Localisation of a fibroblast growth factor and its effect alone and with hydrocortisone on 3T3 cell growth.. Nature.

[OCR_02391] Gospodarowicz D., Weseman J., Moran J. (1975). Presence in brain of a mitogenic agent promoting proliferation of myoblasts in low density culture.. Nature.

[OCR_02405] Heidrick M. L., Ryan W. L. (1971). Adenosine 3',5'-cyclic monophosphate and contact inhibition.. Cancer Res.

[OCR_02400] Heidrick M. L., Ryan W. L. (1970). Cyclic nucleotides on cell growth in vitro.. Cancer Res.

[OCR_02410] Hewitt H. B., Blake E., Proter E. H. (1973). The effect of lethally irradiated cells on the transplantability of murine tumours.. Br J Cancer.

[OCR_02416] Hill R. P., Bush R. S. (1969). A lung-colony assay to determine the radiosensitivity of cells of a solid tumour.. Int J Radiat Biol Relat Stud Phys Chem Med.

[OCR_02420] Jackson R. C., Weber G., Morris H. P. (1975). IMP dehydrogenase, an enzyme linked with proliferation and malignancy.. Nature.

[OCR_02428] Johnson L. D., Abell C. W. (1970). The effects of isoproterenol and cyclic adenosine 3',5'-phosphate on phytohemagglutinin-stimulated DNA synthesis in lymphocytes obtained from patients with chronic lymphocytic leukemia.. Cancer Res.

[OCR_02453] Kolata G. B. (1973). Cyclic GMP: Cellular Regulatory Agent?. Science.

[OCR_02457] LETTRE H. (1952). Some investigations on cell behavior under various conditions; a review.. Cancer Res.

[OCR_02467] Marks F., Grimm W. (1972). Diurnal fluctuation and -adrenergic elevation of cyclic AMP in mouse epidermis in vivo.. Nat New Biol.

[OCR_02482] Munro H. N., Fleck A. (1966). Recent developments in the measurement of nucleic acids in biological materials. A supplementary review.. Analyst.

[OCR_02487] Otten J., Johnson G. S., Pastan I. (1971). Cyclic AMP levels in fibroblasts: relationship to growth rate and contact inhibition of growth.. Biochem Biophys Res Commun.

[OCR_02493] Peters L. J. (1975). Enhancement of syngeneic murine tumour transplantability by whole body irradiation--a non-immunological phenomenon.. Br J Cancer.

[OCR_02499] Peters L. J., Hewitt H. B. (1974). The influence of fibrin formation on the transplantability of murine tumour cells: implications for the mechanism of the Révész effect.. Br J Cancer.

[OCR_02525] ROBINSON K. P., HOPPE E. (1962). The development of blood-borne metastases. Effect of local trauma and ischemia.. Arch Surg.

[OCR_02513] Rixon R. H., Whitfield J. F., Macmanus J. P. (1970). Stimulation of mitotic activity in rat bone marrow and thymus by exogenous adenosine 3'5'-monophosphate (cyclic AMP).. Exp Cell Res.

[OCR_02537] Rudland P. S., Gospodarowicz D., Seifert W. (1974). Activation of guanyl cyclase and intracellular cyclic GMP by fibroblast growth factor.. Nature.

[OCR_02543] Ryan W. L., Heidrick M. L. (1968). Inhibition of cell growth in vitro by adenosine 3',5'-monophosphate.. Science.

[OCR_02562] SELYE H., VEILLEUX R., CANTIN M. (1961). Excessive stimulation of salivary gland growth by isoproterenol.. Science.

[OCR_02573] STOKER M. (1964). REGULATION OF GROWTH AND ORIENTATION IN HAMSTER CELLS TRANSFORMED BY POLYOMA VIRUS.. Virology.

[OCR_02583] SUTHERLAND E. W., RALL T. W. (1958). Fractionation and characterization of a cyclic adenine ribonucleotide formed by tissue particles.. J Biol Chem.

[OCR_02553] Schramm M., Naim E. (1970). Adenyl cyclase of rat parotid gland. Activation by fluoride and norepinephrine.. J Biol Chem.

[OCR_02567] Shaffer B. M. (1975). Secretion of cyclic AMP induced by cyclic AMP in the cellular slime mould Dictyostelium discoideum.. Nature.

[OCR_02647] VOGT P. K., RUBIN H. (1961). Localization of infectious virus and viral antigen in chick fibroblasts during successive stages of infection with Rous sarcoma virus.. Virology.

[OCR_02625] Van Den Brenk H. A., Burch W. M., Kelly H., Orton C. (1975). Venous diversion trapping and growth of blood-borne cancer cells en route to the lungs.. Br J Cancer.

[OCR_02618] Van Den Brenk H. A., Burch W. M., Orton C., Sharpington C. (1973). Stimulation of clonogenic growth of tumour cells and metastases in the lungs by local x-radiation.. Br J Cancer.

[OCR_02594] Van Den Brenk H. A., Kelly H. (1973). Stimulation of growth of metastases by local x-irradiation in kidney and liver.. Br J Cancer.

[OCR_02641] Van Den Brenk H. A., Stone M., Kelly H., Orton C., Sharpington C. (1974). Promotion of growth of tumour cells in acutely inflamed tissues.. Br J Cancer.

[OCR_02606] Van den Brenk H. A., Sharpington C. (1971). Effect of local x-irradiation of a primary sarcoma in the rat on dissemination and growth of metastases: dose-response characteristics.. Br J Cancer.

[OCR_02631] Van den Brenk H. A., Sharpington C., Orton C. (1973). Macrocolony assays in the rat of allogeneic Y-P388 and W-256 tumour cells injected intravenously: dependence of colony forming efficiency on age of host and immunity.. Br J Cancer.

[OCR_02613] Van den Brenk H. A., Stone M. G. (1972). Effects of x-radiation on salivary gland growth in the rat. 3. Relative effects of local irradition on wet weight, protein and nucleic acids of the salivary gland during post-natal growth and secretion and resynthesis of parotid amylase.. Int J Radiat Biol Relat Stud Phys Chem Med.

[OCR_02670] Williams J. F., Till J. E. (1966). Formation of lung colonies by polyoma-transformed rat embryo cells.. J Natl Cancer Inst.

[OCR_02675] Withers H. R., Milas L. (1973). Influence of preirradiation of lung on development of artificial pulmonary metastases of fibrosarcoma in mice.. Cancer Res.

[OCR_02600] van den Brenk H. A., Kelly H. (1974). Potentiating effect of prior local irradiation of the lungs on pulmonary metastases.. Br J Radiol.

